# Multi-Trait Genome-Wide Association Study of Atherosclerosis Detects Novel Pleiotropic Loci

**DOI:** 10.3389/fgene.2021.787545

**Published:** 2022-02-02

**Authors:** Tiffany R. Bellomo, William P. Bone, Brian Y. Chen, Katerina A. B. Gawronski, David Zhang, Joseph Park, Michael Levin, Noah Tsao, Derek Klarin, Julie Lynch, Themistocles L. Assimes, J. Michael Gaziano, Peter W. Wilson, Kelly Cho, Marijana Vujkovic, Christopher J. O’Donnell, Kyong-Mi Chang, Philip S. Tsao, Daniel J. Rader, Marylyn D. Ritchie, Scott M. Damrauer, Benjamin F. Voight

**Affiliations:** ^1^ Department of Surgery, Perelman School of Medicine, University of Pennsylvania, Philadelphia, PA, United States; ^2^ Genomics and Computational Biology Graduate Group, Perelman School of Medicine, University of Pennsylvania, Philadelphia, PA, United States; ^3^ School of Arts and Sciences, University of Pennsylvania, Philadelphia, PA, United States; ^4^ Department of Genetics, University of Pennsylvania, Philadelphia, PA, United States; ^5^ Division of Cardiovascular Medicine, Department of Medicine, University of Pennsylvania Perelman School of Medicine, Philadelphia, PA, United States; ^6^ Corporal Michael J. Crescenz VA Medical Center, Philadelphia, PA, United States; ^7^ VA Boston Healthcare System, Boston, MA, United States; ^8^ Center for Genomic Medicine, Massachusetts General Hospital, Boston, MA, United States; ^9^ Division of Vascular Surgery and Endovascular Therapy, University of Florida School of Medicine, Gainesville, FL, United States; ^10^ Department of Surgery, Massachusetts General Hospital, Boston, MA, United States; ^11^ Program in Medical and Population Genetics, Broad Institute of MIT and Harvard, Cambridge, MA, United States; ^12^ VA Informatics and Computing Infrastructure, VA Salt Lake City Health Care System, Salt Lake City, UT, United States; ^13^ University of Massachusetts College of Nursing and Health Sciences, Boston, MA, United States; ^14^ VA Palo Alto Health Care System, Palo Alto, CA, United States; ^15^ Department of Medicine, Stanford University, Stanford, CA, United States; ^16^ Massachusetts Veterans Epidemiology Research and Information Center, Veterans Affairs Boston Healthcare System, Boston, MA, United States; ^17^ Department of Medicine, Brigham Women’s Hospital, Boston, MA, United States; ^18^ Atlanta VA Medical Center, Decatur, GA, United States; ^19^ Division of Cardiology, Emory University School of Medicine, Atlanta, GA, United States; ^20^ Department of Medicine, University of Pennsylvania, Philadelphia, PA, United States; ^21^ Institute for Translational Medicine and Therapeutics, Perelman School of Medicine, University of Pennsylvania, Philadelphia, PA, United States; ^22^ Department of Pediatrics, University of Pennsylvania, Philadelphia, PA, United States; ^23^ Institute for Biomedical Informatics, Perelman School of Medicine, University of Pennsylvania, Philadelphia, PA, United States; ^24^ Center for Precision Medicine, Perelman School of Medicine, University of Pennsylvania, Philadelphia, PA, United States; ^25^ Department of Systems Pharmacology and Translational Therapeutics, Perelman School of Medicine, University of Pennsylvania, Philadelphia, PA, United States

**Keywords:** peripheral artery disease, atherosclerosis, multi-trait analyses, GWAS—genome-wide association study, pleiotropy

## Abstract

Although affecting different arterial territories, the related atherosclerotic vascular diseases coronary artery disease (CAD) and peripheral artery disease (PAD) share similar risk factors and have shared pathobiology. To identify novel pleiotropic loci associated with atherosclerosis, we performed a joint analysis of their shared genetic architecture, along with that of common risk factors. Using summary statistics from genome-wide association studies of nine known atherosclerotic (CAD, PAD) and atherosclerosis risk factors (body mass index, smoking initiation, type 2 diabetes, low density lipoprotein, high density lipoprotein, total cholesterol, and triglycerides), we perform 15 separate multi-trait genetic association scans which resulted in 25 novel pleiotropic loci not yet reported as genome-wide significant for their respective traits. Colocalization with single-tissue eQTLs identified candidate causal genes at 14 of the detected signals. Notably, the signal between PAD and LDL-C at the *PCSK6* locus affects *PCSK6* splicing in human liver tissue and induced pluripotent derived hepatocyte-like cells. These results show that joint analysis of related atherosclerotic disease traits and their risk factors allowed identification of unified biology that may offer the opportunity for therapeutic manipulation. The signal at *PCSK6* represent possible shared causal biology where existing inhibitors may be able to be leveraged for novel therapies.

## Introduction

Atherosclerotic vascular disease is a leading cause of death worldwide (Lozano et al., 2012; Kobiyama and Ley, 2018; Virani et al., 2020) and can affect multiple arterial territories. Although clear differences in disease pathobiology exist (Lin et al., 2013), epidemiological analyses have shown both coronary artery disease (CAD) and peripheral artery disease (PAD) share similar risk factors and frequently co-occur (Ozkaramanli Gur et al., 2018; Klarin et al., 2019; Sundaram et al., 2020). These risk factors include dyslipidemia, obesity, hypertension, diabetes, and tobacco use (Criqui and Aboyans, 2015). PAD patients with concomitant CAD are known to experience more extensive and aggressive disease (Hussein et al., 2011).

The genetics of CAD have been well characterized and a number of genome-wide association studies (GWAS) have identified over 200 genetic risk loci with robust connections to CAD (Khera and Kathiresan, 2017; Van Der Harst and Verweij, 2018; Koyama et al., 2020). For most loci, however, underlying mechanisms by which these loci influence CAD risk remains unclear. Although PAD has been less intensively studied, recent work has identified 21 total risk loci associated with PAD risk (Matsukura et al., 2015; Klarin et al., 2019). Genetic correlation studies have demonstrated a high degree of shared genetic architecture between CAD and PAD (LD-score regression-based genetic correlation r_g_ = 0.67) (Purcell et al., 2007). This genetic correlation, based on shared pathobiology, can be leveraged to identify novel pleiotropic genetic architecture common to both disease traits (Zhao et al., 2017; Baselmans et al., 2019).

The development of statistical approaches for multi-trait GWAS meta-analysis has facilitated joint analyses of traits with substantial evidence for a common pathophysiological basis to elucidate shared genetic etiology (Klarin et al., 2019). Furthermore, correlated causal risk factors can also be included in these multi-trait GWAS analyses to provide insight on their shared genetic pathways (Holmes et al., 2015; Zhao et al., 2017; Riaz et al., 2018; Siewert and Voight, 2018; Larsson et al., 2020). Our previous work has analyzed CAD pairwise with secondary traits to understand shared genetic etiology to successfully identify new risk loci (Zhao et al., 2017; Siewert and Voight, 2018). Yet, there have been no studies which evaluate atherosclerosis endpoints jointly with multiple cardiometabolic causal risk factors for discovery and quantitative interpretation.

In this study, we performed a series of N-weighted multivariate genome-wide-association meta-analyses (N-GWAMA) (Baselmans et al., 2019) using different combinations of nine atherosclerotic or atherosclerosis risk factor traits, and identified 31 unique pleiotropic loci not previously associated with any analyzed trait combination. We subsequently used single-tissue expression quantitative trait loci (eQTL) colocalization analysis at these loci to identify candidate causal genes and their tissue site of action. Some of these causal gene candidates have potential opportunities for drug target repurposing to treat atherosclerotic vascular disease, including *PCSK6*. Ultimately, this study provides a better understanding of biological pathways underlying atherosclerosis to inform future therapeutic development.

## Methods

This study was approved by the U.S. Department of Veterans Affairs Central Institutional Review Board. All participants gave written informed consent for study participation.

### Genetic Association Data

We collected the summary statistics from the largest published GWAS to maximize our power for novel discovery. PAD summary statistics were the European ancestry subjects from the recent VA Million Veteran Program analysis which consisted of 24,009 PAD cases and 150,983 PAD controls (Klarin et al., 2019). These data can be accessed from dbGAP (phs001672). CAD data were taken from CARDIoGRAMplusC4D combined with the United Kingdom BioBank (UKBB) (Van Der Harst and Verweij, 2018) and consisted of 122,733 CAD cases and 424,528 CAD controls. Data for body mass index (BMI) (meta-analysis of GIANT and UKBB; 806,834 individuals; (Yengo et al., 2018)), type 2 diabetes (T2D) (meta-analysis of consortia; 228,499 cases and 1,178,783 controls; (Vujkovic et al., 2020)), smoking initiation (smoking) (UKBB; 462,690 individuals; (Wootton et al., 2020)), and 4 lipid traits (meta-analysis of MVP and GLGC data; 723,000 participants; (Klarin et al., 2018)). Access urls for all data obtained from the public domain are provided in Supplementary Table S1.

### N-GWAMA Multi-Trait GWAS

Using the summary statistics from publicly available single-trait GWAS (Supplementary Table S1), we performed 15 N-GWAMA (Baselmans et al., 2019) multi-trait GWAS centered around PAD, CAD, and the following atherosclerotic risk factor traits: BMI, smoking, T2D, LDL-C, HDL-C, TC, and TG. Full details are provided in Supplementary Methods. Briefly, we first performed a bivariate GWAS for PAD and CAD followed by a series of trivariate GWAS combining PAD, CAD, and one of seven correlated traits that represented traditional atherosclerotic risk factors. We also performed a series of bivariate GWAS between PAD and these seven traits individually, given that a series of bivariate GWAS between CAD and most of these seven traits has already been performed (Siewert and Voight, 2018).

**FIGURE 1:**
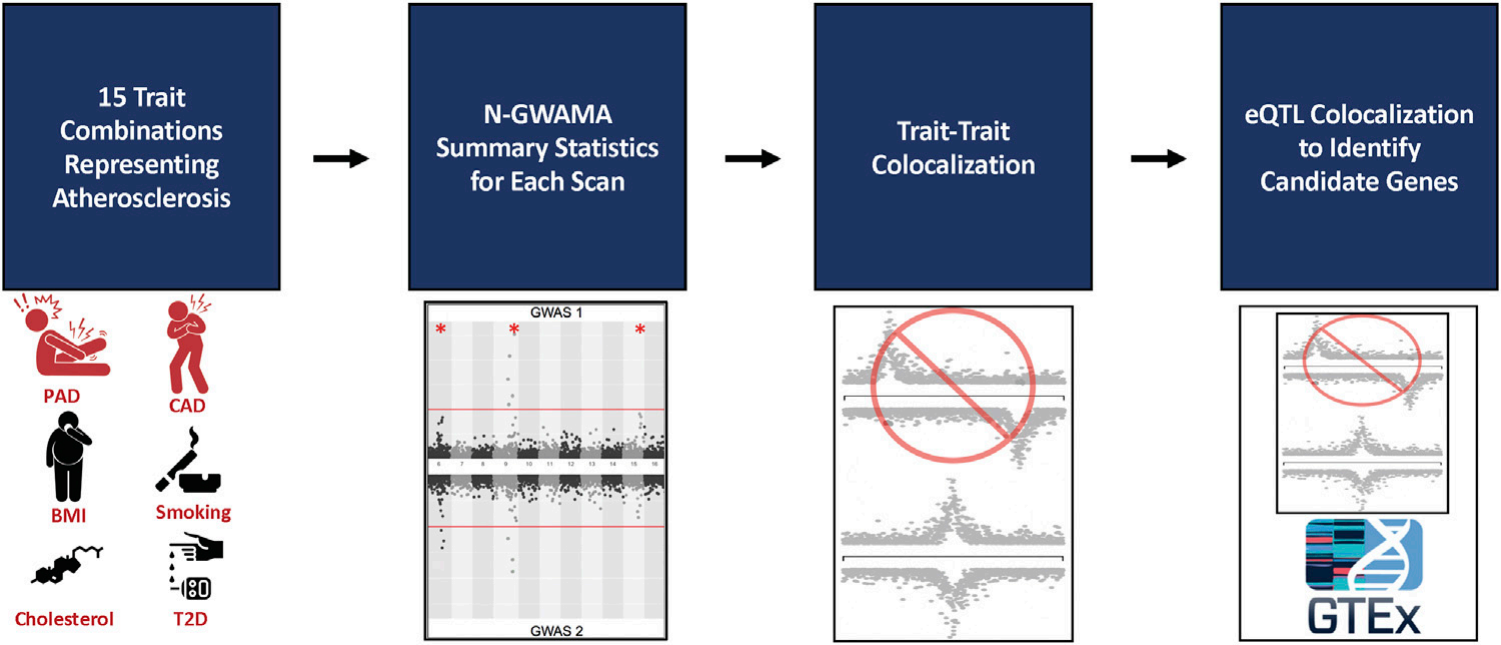
Flowchart of multi-trait analysis and candidate gene results. 9 traits were analyzed in 15 different bivariate and trivariate scans that best represented atherosclerosis. The summary statistics from all scans were filtered by single trait *p*-values and loci within 500 kb or in LD (EUR *r* ^2^ > 0.21 kG EUR) with the known trait being tested according to the GWAS Catalog, resulting in 150 unique loci. Trait-to-trait colocalization with a threshold of a conditional posterior probability of colocalization >0.8 was performed to ensure evidence of a shared causal SNP between each trait. The resulting 31 unique loci were run through single tissue eQTL analysis using GTEx v8 to identify candidate causal genes and tissues for each locus. 34 unique genes were identified among 14 loci.

Each N-GWAMA multi-trait GWAS resulted in a set of independent loci represented by a sentinel SNP. We defined an independent locus as the genomic region that includes all variants within 1 megabase (Mb) of the sentinel SNP and any other variants that were in linkage diseqilibrium (LD) of *r* ^2^ > 0.2 with the sentinel SNP using the 1,000 Genomes European ancestry cohort (1 kG EUR) (Purcell et al., 2007). We then applied a series of filters to remove loci that were not plausibly pleiotropic, or did not represent novel associations. To ensure there was evidence a locus was pleiotropic and that a single trait was not driving the association, we required that the sentinel SNP was at least nominally associated (*p* < 5 × 10^−3^) with all the individual traits involved in the multi-trait analysis. We also required that none of the variants at an independent locus were previously associated with any of the traits used in the N-GWAMA multi-trait GWAS by applying two filters. First, we required each sentinel SNP was not genome-wide significant for any of the individual traits (*p* > 5 × 10^−8^). It was also necessary that none of the SNPs at the independent locus were previously reported to be genome-wide significant for any of the individual traits involved in the multi-trait analysis in the GWAS Catalog (Buniello et al., 2019) (Figure 1). Finally, we excluded loci in the HLA region from these experiments due to the difficulty of interpreting the independent signals of these loci. Code for the pipeline is available at: https://github.com/Bellomot/Athero_NGWAMA_Multitrait_GWAS.

Given that we performed 15 multi-trait GWAS for combinations of related traits, we next implemented a multiple testing correction procedure to assess significance. Due to the high correlation between each of the multi-trait GWAS we performed, a Bonferroni correction (*p* < 3.3 × 10^−9^) for each trait combination test is conservative. Thus, we constructed a null distribution Z-score sampling strategy to estimate an *α* = 0.05 *p*-value threshold given the set of N-GWAMA multi-trait GWAS that we performed. Under the assumption that the correlation of the Z-scores across the entire genome that resulted from the N-GWAMA multi-trait GWAS are a reasonable estimate of the correlation in the multivariate null distribution of Z-scores, we can use the correlation matrix of the Z-scores from the 15 multi-trait GWAS to model the 15-dimentional multivariate standard normal distribution that is the theoretical null distribution of these results.

To get this estimate of the appropriate *α* = 0.05 *p*-value threshold, we first drew 10,000 sets of 1 million samples from a 15-dimentional multivariate normal distribution centered at the origin, and used the correlation between the Z-scores of all the SNPs that were tested across all 15 N-GWAMA multi-trait GWAS as the correlation matrix (Supplementary Table S2). We kept the most extreme Z-score from each of the 10,000 sets and then identified the 95th percentile of the most extreme Z-scores as our *α* = 0.05 threshold. We defined experiment-wide significance as the 95th percentile Z-score of 5.87, which corresponds to a *p*-value of 4.3 × 10^−9^.

### Trait-Trait Colocalization

For each multi-trait GWAS, we assessed the evidence of a shared causal variant at each significant locus by performing colocalization analysis between the trait signals using COLOC for bivariate GWAS and MOLOC for trivariate GWAS (Giambartolomei et al., 2014; Giambartolomei et al., 2018) (Supplementary Table S3). For this analysis, we applied a 500 kilobases (Kb) window (+/− 250 Kb) around the sentinel SNP. A conditional probability of colocalization is defined as the posterior probability of colocalization conditioned on the presence of a signal for each trait. For example, when using COLOC, this would be represented as the posterior probability of hypothesis four (PP4) over the sum of the posterior probabilities of hypothesis three (PP3) and PP4 (PP4/(PP3 + PP4)) (Giambartolomei et al., 2014). A probability of ≥0.8 was considered of interest. Loci that had a conditional probability of colocalization >0.5 and <0.8 were visually inspected using LocusZoom plots (Supplementary Table S4). If the LD structure suggested additional associations unlinked to the sentinel SNP, approximate conditional analysis was performed (see details on Approximate Conditional Analysis below).

### Single-Tissue Gene Expression Colocalization

We performed single-tissue colocalization analysis to prioritize candidate causal genes implicated in each N-GWAMA multi-trait GWAS using RNA-seq data obtained from the Genotype-Tissue Expression (GTEx) project (Giambartolomei et al., 2014). We identified the list of genes and tissues for which each N-GWAMA sentinel SNP was a significant single-tissue eQTL in any GTEx v8 tissue as reported in the “.signif_variant_gene_pairs.txt.gz” files available from the GTEx Portal (Supplementary Table S5). We then performed colocalization between either CAD or PAD, as determined by which trait had the most significant sentinel SNP at each locus, and each single-tissue eQTL signal (Giambartolomei et al., 2014). We selected the window of colocalization to be 500 Kb spanning the sentinel SNP. Similar to trait-trait colocalization analysis, our threshold to classify the traits as colocalized was a conditional probability of colocalization (PP4/(PP3 + PP4)) ≥ 0.8. We visually inspected LocusZoom plots for loci where colocalization analysis resulted in a conditional probability of colocalization <0.8 but >0.5 and performed approximate conditional analysis when the LD structure suggested possible allelic heterogeneity (see details on Approximate Conditional Analysis below).

### Approximate Conditional Analysis

For each locus that showed evidence of multiple independent signals, we performed approximate conditional analysis on variants that appeared to be associated with the trait of interest independently of the sentinel SNP (Supplementary Table S6). This analysis was necessary given that the presence of multiple associated variants in a region violates the assumptions of COLOC (Giambartolomei et al., 2014). Potential nearby association signals were identified using LocusZoom plots and the LDlink LDassoc tool between sentinel and putative distinctive signal variant quantified using LDlink (Pruim et al., 2010; Machiela and Chanock, 2018). We performed approximate conditional analysis using GCTA-COJO with 1,000 Genome Project data (European samples, *n* = 503) as a reference panel (Yang et al., 2011; 1000 Genomes Project Consortium et al., 2012). We conditioned the sentinel SNP on the most associated variant for each potential confounding signal identified at the locus. We then repeated the colocalization experiment on the locus using the conditional variant *p*-values. A full list of traits, the sentinel SNPs, and the conditioned variants for each conditional analysis are provided in the supplement (Supplementary Table S6).

### Splicing Quantitative Trait Locus Colocalization

**FIGURE 2:**
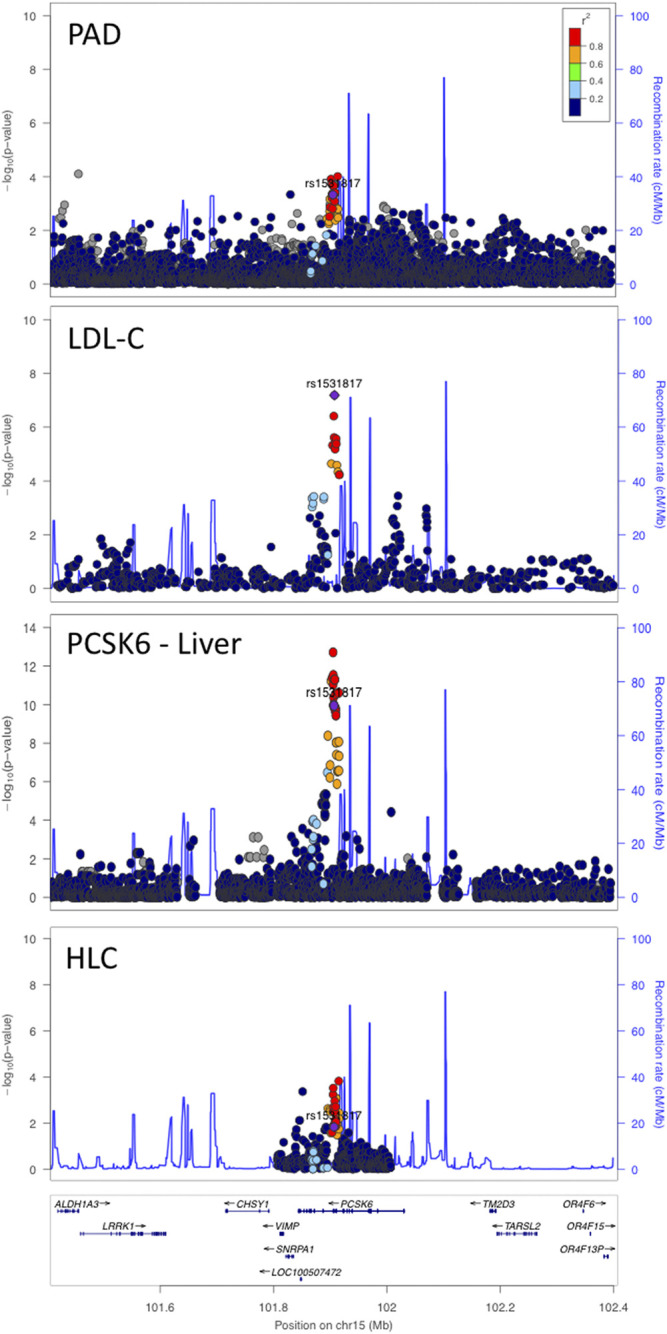
*PCSK6* locus with a sentinel SNP of rs1531817. Pleiotropic signal between PAD and LDL-C with an sQTL for *PCSK6* in liver tissue. This locus also colocalized with hepatocyte-like cells (HLCs) *in vitro*.

We performed a colocalization analysis between the PAD signal at the *PCSK6* locus and the GTEx v8 liver tissue splicing quantitative trait locus (sQTL) signal with the intron ID: 101365044:101366196:clu_14775. We also identified this intron signal in the Phenotyping Lipid traits in iPS derived hepatocytes Study (PhLiPS) hepatocyte-like cell (HLC) sQTL data by lifting over the start and stop of this intron to hg19 (101905249:101906401) (Gawronski et al., 2019). We then performed colocalization analysis between the HLC sQTL signal and the PAD signal as well as the HLC sQTL signal and the GTEx v8 liver tissue sQTL signal (Figure 2).

## Results

### Multi-Trait GWAS Analysis Results

We first calculated the genetic correlation between PAD and CAD and the seven atherosclerosis risk factors (BMI, smoking, T2D, LDL-C, HDL-C, TC, and TG) using the summary statistics files for these GWAS using LD score regression (Supplementary Figure S1; Supplementary Table S7). We then performed 15 N-GWAMA scans centered around PAD and CAD to detect novel loci not previously reported as genome-wide significant for any of their respective traits (Supplementary Table S8). A total of 150 sentinel SNPs were multivariate genome-wide significant with all single trait *p*-values between <5 × 10^−3^ and >5 × 10^−8^ (Supplementary Table S3). Of these sentinel SNPs, 31 were nominal genome-wide significant (multi-trait *p* < 5 × 10^−8^) and met our trait-to-trait colocalization criteria, and thirteen were experiment-wide significant (multi-trait *p* < 4.3 × 10^−9^) and met our trait-to-trait colocalization criteria. If we had used the Bonferroni correction threshold, only one locus, *NUP85* (*p* = 3.55 × 10^−9^), would change from experiment-wide significant to genome-wide significant. The nominal genome-wide significant sentinel SNPs represent 25 independent loci and 11 experiment-wide significant independent loci (>1 Mb from any of our other reported loci, Table 1, Supplementary Figures S2–S34). Fourteen of the nominal genome-wide significant sentinel SNPs and eight experiment-wide significant sentinel SNPs colocalized with one or more single-tissue eQTLs (Supplementary Table S5). Finally, five loci colocalized with eQTLs for genes that have been implicated in atherosclerosis by previous studies.

**TABLE 1:** Atherosclerosis trait N-GWAMA analysis and results. Trait 3 *p* value will have a value of NA if there were only 2 traits analyzed. Conditional posterior probability represents the probability of the trait-to-trait colocalization analysis (e.g., PP4/(PP3 + PP4)).

Trait 1, Trait 2, Trait 3	Locus name	Sentinel SNP	Chr	Position GRCh37	Effect	Other allele	Direction of effect for each trait	Effect allele frequency	Multivariate *p* value	Trait 1 *p* value	Trait 2 *p* value	Trait 3 *p* value
PAD, CAD, T2D	SATB1	rs9845140	3	18728878	C	A	−/−/−	0.27	2.06E-11	2.59E-05	8.18E-06	2.09E-05
PAD, CAD, HDL	LRCH1	rs9526214	13	47237213	T	C	−/−/−	0.24	3.38E-11	6.17E-06	7.06E-05	8.31E-04
PAD, CAD	CTGE1/CTGE2	rs948386	18	19998810	G	C	−/−	0.42	4.10E-11	2.20E-05	1.49E-07	NA
PAD, LDL	PCSK6	rs1531817	15	101906737	C	A	+/+	0.68	3.15E-10	4.72E-04	6.48E-08	NA
PAD, CAD, TG	SAMD8	rs9299525	10	76878025	G	A	−/−/−	0.58	5.73E-10	2.35E-05	2.15E-05	5.30E-04
PAD, CAD	NFAT5	rs1364063	16	69588572	T	C	+/+	0.59	6.63E-10	6.27E-08	2.17E-05	NA
PAD, CAD, T2D	SPP2C	rs55660209	17	43932173	T	C	−/−/−	0.79	6.68E-10	7.41E-04	5.18E-04	7.41E-06
PAD, CAD, BMI	PNPLA3	rs2076211^a^	22	44329078	C	T	+/+/+	0.84	7.56E-10	2.47E-03	4.55E-04	3.13E-06
PAD, CAD, SMK	HMBS	rs1006195	11	118958869	G	T	−/−/−	0.60	1.92E-09	2.97E-04	1.03E-06	3.90E-03
PAD, CAD	SATB1	rs9826966	3	18737796	A	G	−/−	0.27	2.14E-09	2.53E-05	4.01E-06	NA
PAD, TG	ATAD5	rs7342938	17	29189830	A	G	+/+	0.88	2.45E-09	1.86E-04	2.47E-07	NA
PAD, T2D	ARL17	rs2458203	17	44336651	T	C	−/−	0.67	3.11E-09	3.51E-03	8.36E-08	NA
PAD, TG	NUP85	rs2291031	17	73228173	C	T	+/+	0.82	3.55E-09	2.50E-03	7.45E-08	NA
PAD, T2D	ZN536	rs73022871	19	30990705	C	G	+/+	0.85	4.70E-09	2.48E-03	1.29E-07	NA
PAD, LDL	BPTF	rs12602912	17	65870073	C	T	−/−	0.79	5.24E-09	1.20E-06	2.42E-05	NA
PAD, BMI	OPN5	rs9381618	6	47780081	T	C	−/−	0.72	6.35E-09	1.24E-05	9.57E-07	NA
PAD, TG	OR4CD	rs10839321	11	49670562	T	C	−/−	0.91	6.51E-09	3.86E-03	1.08E-07	NA
PAD, CAD, SMK	ZN268	rs61960706	12	133777822	G	A	−/−/−	0.74	7.18E-09	2.56E-03	3.27E-05	6.10E-04
PAD, CAD	VDAC2	rs7088974	10	76891096	T	C	−/−	0.57	7.73E-09	1.26E-05	2.08E-05	NA
PAD, TG	ATG7	rs2606736	3	11400249	C	T	−/−	0.38	8.21E-09	2.59E-03	1.73E-07	NA
PAD, CAD	CBPC2	rs11602961	11	47727748	C	T	−/−	0.94	8.83E-09	5.81E-04	2.07E-06	NA
PAD, T2D	L2HDH	rs72683923	14	50735947	T	C	+/+	0.98	9.75E-09	7.24E-05	1.24E-06	NA
PAD, T2D	MPPD2	rs1765131	11	30404538	G	C	+/+	0.65	1.05E-08	1.45E-04	1.43E-06	NA
PAD, TC	S4A8	rs9795910^a^	12	51795623	A	G	+/+	0.62	1.83E-08	1.81E-03	1.04E-06	NA
PAD, TC	SORCS3	rs11599236	10	106454672	T	C	+/+	0.59	2.38E-08	3.66E-05	1.24E-05	NA
PAD, BMI	LTOR3	rs185238112	4	100801033	C	T	−/−	0.94	2.49E-08	1.59E-03	7.91E-07	NA
PAD, SMK	KPCD1	rs10149845	14	30177079	C	T	−/−	0.59	3.00E-08	3.92E-05	2.10E-05	NA
PAD, T2D	SATB1	rs4269101	3	18763543	T	G	−/−	0.28	3.59E-08	1.95E-05	5.78E-06	NA
PAD, LDL	S4A8	rs9795910^a^	12	51795623	A	G	+/+	0.62	3.86E-08	1.81E-03	2.72E-06	NA
PAD, BMI	CDKL1	rs11570792	14	50847010	C	T	+/+	0.95	4.13E-08	1.39E-03	1.39E-06	NA
PAD, CAD, TG	TMM18	rs2867113	2	651,365	G	A	+/+/+	0.82	4.67E-08	1.14E-03	1.50E-04	1.15E-03

a indicates that the sentinel SNP was detected in another trait combination scan.

Loci in gray met the experiment-wide significance threshold (*p*-value < 4.3 × 10^–9^). BMI, body mass index; CAD, coronary artery disease; Chr, chromosome; HDL-C, high density lipoprotein; LDL-C, low density lipoprotein; PAD, peripheral artery disease; SMK, smoking; T2D, type 2 diabetes; TC, total cholesterol; TG, triglycerides.

We noted that two of our signals that exceeded multi-trait experiment-wide significance mapped to a previously established locus for several cardiometabolic traits. That signal was tagged by rs2076211, and associated with PAD, CAD, and BMI (bivariate *p* = 7.6 × 10^−10^) or PAD, CAD, and LDL (bivariate *p* = 4 × 10^−9^). This variant mapped to the nearby gene *PNPLA3*, a well-established locus associated for non-alcoholic fatty liver disease (Speliotes et al., 2011), multiple liver enzymes measures (Yuan et al., 2008), hemotological traits (Kamatani et al., 2010), sex-hormone binding globulin levels (Ruth et al., 2020), and T2D (Mahajan et al., 2018). Our sentinel SNP that tagged both multi-trait signals was in strong LD with rs738409 (*r* ^2^ = 0.73 1 kG EUR), the previously reported sentinel variant associated with these additional traits. A previous multi-trait scan for T2D and LDL reported strong association for both traits at this locus (Klimentidis et al., 2020). However, association with this locus and atherosclerotic disease (PAD, CAD) or to BMI to our knowledge has not been previously reported, but are compelling given the extensive pleiotropy for atherosclerotic causal risk factors here.

### *PCSK6* Locus

We detected a signal that exceeded multi-trait experiment-wide significance with PAD and LDL-C (bivariate *p* = 3.2 × 10^−10^) at the *PCSK6* locus. A rare coding variant in this region has been reported to associate with LDL-C (Klimentidis et al., 2020; Sinnott-Armstrong et al., 2021), however, the coding variant (NP_002561.1:p.Thr964Met, rs34631529) and our sentinel SNP (rs1531817) are not in linkage disequilibrium (*r* ^2^ = 0.0086 1 kG EUR) based on data from the 1,000 Genomes Project (Purcell et al., 2007), indicating that we detected a different signal at this locus. To further differentiate whether our signal was novel, we performed an additional conditional analysis on the coding variant rs34631529 in PAD data without any notable changes in the *PCSK6* locus signal (Supplementary Figure S36). We further note that previous GWAS have found that variants at this locus are associated with inflammatory markers (Iyengar et al., 2015; Hackinger et al., 2018; Nath et al., 2019; Folkersen et al., 2020; Richardson et al., 2020; Ruotsalainen et al., 2021).

To better understand how genetic variation at the *PCSK6* locus influences circulating lipid levels, we investigated the association of the bivariate sentinel SNP at this locus in the publicly available GWAS of NMR lipid subfractions: extra-small subfrations (XS), extra-large subfractions (XL), HDL, intermediate density lipoprotein (IDL), LDL, and very-low density lipoprotein (VLDL) (Kettunen et al., 2016). We found our sentinel SNP (rs1531817) had a nominal association with medium VLDL particles (β = 0.03, SE = 0.01, *p* = 9 × 10^−3^), total lipids in medium VLDL (β = 0.03, SE = 0.01, *p* = 0.02), TG in medium VLDL (β = 0.02, SE = 0.01, *p* = 0.03), serum TG (β = 0.02, SE = 0.01, *p* = 0.03), and TG in large VLDL (β = 0.02, SE = 0.01, *p* = 0.03).

Our sentinel SNP was a sQTL for *PCSK6* in GTEx v8 liver tissue (Figure 2). To identify a potential experimental model of this splicing change, we searched for this sQTL in PhLiPS HLC summary data (PhLiPS Study) (Pashos et al., 2017). The signal at *PCSK6* colocalized with an sQTL in these data as well (Figure 2), which suggests that derived hepatocyte-like cells could be a good model for further studied of the effect of this locus on LDL-C and PAD risk.

### *SORCS3* Locus

We detected a nominal genome-wide significant signal with PAD and TC (bivariate *p* = 2.4 × 10^−8^) at the *SORCS3* locus rs11599236 (Table 1). This locus was previously observed to be genome-wide significant in GWAS studies of mood disorders (Howard et al., 2018; Ward et al., 2020) (Supplementary Table S4). This signal colocalized with *SORCS3* mRNA expression in pituitary tissue (Supplementary Figure S34). The opposite direction of effect was noted for both traits and the gene-tissue pair: decreased *SORCS3* associated with increased PAD and TC (β = 0.31, SE = 0.05, *p* = 5.5 × 10^−8^).

### Other Candidate Genes Indicated With Known Atherosclerotic Biology

We detected a trivariate GWAS signal with PAD, CAD, and smoking (trivariate *p* = 1.9 × 10^−9^) at the *HMBS* locus rs1006195 (Table 1). This variant was genome-wide significant in previous GWAS studies for several cardiometabolic traits including Apolipoprotein A1 levels, waist-hip ratio, BMI, fat mass percentage, HDL-C, and T2D (Turcot et al., 2018; Van Der Harst and Verweij, 2018; Pulit et al., 2019; Richardson et al., 2020; Zhu et al., 2020). This pleiotropic signal colocalized with *HMBS* and *VPS11* mRNA expression in several tissues (Supplementary Figure S14; Supplementary Table S5). *HMBS* (β = 0.28, SE = 0.03, *p* = 4.1 × 10^−23^) demonstrated the same direction of effect with PAD, CAD, and smoking, indicating that increased *HMBS* expression is associated with increased PAD, CAD, and smoking risk. *VPS11* (β = −0.25, SE = 0.03, *p* = 6.0 × 10^−15^) demonstrated the opposite direction of effect in all tissue except skeletal muscle and the left ventricle of the heart, meaning increased *VPS11* expression in skeletal muscle and left ventricle is associated with increased PAD, CAD, and smoking risk.

We also detected a nominal genome-wide significant signal with PAD and CAD (bivariate *p* = 7.8 × 10^−9^) at the *VDAC2* locus rs7088974 (Table 1). Variants at this locus have been found to be associated with BMI in previous GWAS(Pulit et al., 2019) (Supplementary Table S4). Although variants near this locus have also been associated with smoking behavior, our results suggest that the locus we detected is independent of smoking behavior (Liu et al., 2019) (Supplementary Figure S35). This signal colocalized with *VDAC2* mRNA expression in multiple vascular tissues relevant to atherosclerosis, including aorta and tibial artery (Supplementary Table S5). The direction of effect in all tissue was opposite to the direction of effect for PAD and CAD: the allele associated with increased *VDAC2* expression (EA = C, EAF = 0.57) is associated with decreased PAD and CAD (β = −0.14, SE = 0.02, *p* = 7.7 × 10^−9^).

Finally, we detected a trivariate signal with PAD, CAD, and HDL-C (trivariate *p* = 3.4 × 10^−11^) at the *LRCH1* locus rs9526214 (Table 1). This locus had evidence of allelic heterogeneity when we reviewed the regional association plots, which led us to perform approximate conditional analyses on the pleiotropic signal sentinel SNP rs9316223 and the resulting conditional probability of colocalization met our criteria (Supplementary Table S5). This locus has been found to be genome-wide significant in previous GWAS studies for platelets, systolic blood pressure, and stroke (Evangelou et al., 2018; Malik et al., 2018) (Supplementary Table S4). This signal colocalized with *LRCH1* mRNA expression in tibial artery, whole blood, and other tissues (Supplementary Figure S12; Supplementary Table S5). The opposite direction of effect was noted for all three traits and the gene tissue pair: the allele associated with increased *LRCH1* was also associated with decreased PAD, CAD, and HDL-C (β = −0.09, SE = 0.02, *p* = 1.3 × 10^−8^).

## Discussion

To advance our understanding of the genetic etiology of atherosclerosis, different combinations of nine known atherosclerotic or atherosclerosis risk factor traits were used to perform 15 N-GWAMA scans which resulted in 25 unique novel pleiotropic loci (Figure 1). Colocalization with single-tissue eQTLs identified 34 candidate causal genes across 14 of the detected signals. Five of these loci had candidate causal genes previously associated with atherosclerosis through other studies. While candidate causal genes remain elusive for the remaining loci, the patterns of association represent physiology that appears compelling. For example, our top association at the *SATB1* locus was modestly associated with CAD, PAD, and T2D in the same direction of effect, implying that a perturbation informed by the human genetics data might be expected to be ameliorative for all three traits. Functional work to elucidate causal variant, genes, and mechanism at these loci thus may provide new insights into the etiological pathways for this collection of disease endpoints.

### PCSK6 Activity Effects Lipid Levels, Plaque Formation and Stability

We identified a signal at the *PCSK6* locus that has a bivariate association with PAD and LDL-C and provide strong evidence in support of *PCSK6* as the causal gene at the *PCSK6* locus. PCSK6 is a calcium-dependent serine endoprotease that cleaves proteins to active and inactive forms depending on the target protein (Kiefer et al., 1991).

There is convincing experimental evidence to suggest that PCSK6 directly influences plaque development and plaque stability. Smooth muscle cell migration in injured arteries is facilitated by cytokine induced *PCSK6* expression that activates matrix metalloproteinases (MMP14/MMP2) (Perisic et al., 2013). This smooth muscle cell mechanism may explain the association of *PCSK6* with carotid intima-media thickness in a candidate gene study (Rykaczewska et al., 2020). The sentinel SNP associated with maximum progression of carotid intima-media thickness was the same as the sentinel SNP identified in our bivariate scan between LDL-C and PAD. There is also evidence that PCSK6 activates MMP9, which enhances degredation of the extra cellular matrix and thus promotes plaque instability (Li et al., 2020; Testa et al., 2021).

Our data suggest *PCSK6* also influences lipid metabolism, a known upstream cause of atherosclerotic progression. This is in agreement with the known role of PCSK6 in lipid metabolism, where it cleaves and inactivates endothelial lipase (EL) and lipoprotein lipase (LPL) (Jin et al., 2005), which can lead to hyperlipidemia (Choi and Korstanje, 2013). This finding is further supported by the recently reported rare coding variant in *PCSK6* associated with decreased LDL-C (Klimentidis et al., 2020; Sinnott-Armstrong et al., 2021).

It remains to be determined how changes in PCSK6 activity result in altered lipid metabolism and risk of PAD. It is possible that changes in PCSK6 activity alter lipid metabolism and therefore have downstream effects on PAD or altered PCSK6 activity may effect LDL-C and PAD separately through independent mechanisms. In all likelihood, it is a combination of LDL-C dependent and independent mechanisms that link PCSK6 to PAD.

From a drug targeting standpoint, we would expect that inhibiting PCSK6 reduces LDL-C levels and PAD risk. There are several non-FDA approved, non-specific PCSK6 inhibitors that influence lipid metabolism: alpha1-antitrypsin Portland (alpha1-PDX) (Gauster et al., 2005), profurin (Jin et al., 2007), Pf-pep (Byun et al., 2010), and dicoumarols (DC), specifically DC2 (Komiyama et al., 2009).

### SORCS3 Influences Energy Metabolism

We identified a novel pleiotropic association at the *SORCS3* locus between PAD and TC. Colocalization experiments support *SORCS3* as the causal gene at this locus. SORCS3 is a type I transmembrane protein that is a member of the Vps10p receptor family (Hermey, 2009). The post-synaptic sorting receptor SORCS3 is highly expressed in the hippocampus and binds NGF and PDGF-BB to modulate several signal transduction pathways involved in neuronal activity (Hermey, 2009; Christiansen et al., 2017). A recent knockout mouse study has shown that even loss of one *Sorcs3* allele decreases lipid metabolism as a source of energy and increases adiposity (Subkhangulova et al., 2018). The proposed mechanism for this occurrence is that loss of *SORCS3* expressed in agouti-related peptide (AGRP) neurons leads to unchecked production of circulating AGRP (Henry et al., 2015). ARGP is well described to cause enhanced food intake, decreased locomotor activity, decreased use of lipids as metabolic fuel, and overall increased adiposity (Maier et al., 2018; Beutler et al., 2020). One limitation of GTEx data is a very small sample size of hypothalamus data, our data show that reduced expression of *SORCS3* in the pituitary gland is related to high levels of TC and an increased overall risk for PAD, potentially through increased circulating AGRP diverting energy metabolism away from lipid sources.

### Limitations

We acknowledge there are several limitations to this study. First, there is sample overlap between several of our single trait summary statistics files. The N-GWAMA method attempts to account for this, but if the correction for the overlap was insufficient this could inflate our false discovery rate. Second, in using the null Z-score resampling strategy to estimate the appropriate multiple testing correction, we assume that the correlation of the Z-scores across the entire genome that resulted from the N-GWAMA multi-trait GWAS are a reasonable approximation of the correlation between the multivariate null distribution of Z-scores. This assumption could be broken due to highly associated SNPs leading to an altered correlation matrix compared to the true null distribution and the Z-scores having an inflated variance from confounders that may not have been corrected for by LDSC. Finally, we selected nine atherosclerotic and cardiometabolic traits based on conventional relationships with atherosclerosis; however, there are likely multifactorial and multidirectional relationships within this group of traits. It is possible that some of the novel loci represent the interaction between traits instead of the intended representation of atherosclerosis as we have interpreted it. It is also possible that including other sets of cardiometabolic risk factors may identify additional novel loci.

## Conclusion

We have shown that publicly available GWAS data can be leveraged to perform multi-trait scans with N-GWAMA methods to identify novel loci that unify atherosclerosis. In this study, 25 nominal genome-wide significant loci were associated jointly with PAD and other atherosclerotic traits. These loci may represent novel genetic etiologies of atherosclerosis. A total of 34 candidate causal genes were identified across 14 novel pleiotropic loci and among those, *PCSK6* represents possible causal biology with known inhibitors that has large potential to be a therapeutic target for atherosclerosis. These results highlight the biological underpinnings of atherosclerosis and the potential to develop non-invasive medical treatments for atherosclerosis.

## Data Availability

The data analyzed in this study is subject to the following licenses/restrictions: The GWAS and GTEx eQTL data are publicly available. GWAS Summary Statistics also in Supplementary Table S1: PAD: dbGAP accession code phs001672.v2.p1; CAD: Mendeley doi: 10.17632/gbbsrpx6bs.1; Lipids: MVP, dbGAP accession codes phs001672.v4.p1, pha004828.1, pha004831.1, pha004837.1, pha004834.1; GLGC, http://csg.sph.umich.edu/willer/public/lipids/; T2D: dbGAP under accession number phs001672.v3.p1; BMI: https://portals.broadinstitute.org/collaboration/giant/index.php/GIANT_consortium_data_files#20; Smoking: https://data.bris.ac.uk/data/dataset/10i96zb8gm0j81yz0q6ztei23d; GTEx v8 data is available at: https://gtexportal.org/home/datasets Additional data that support the findings of this study are available on request from the coauthor (SD); these data are not publicly available due to U.S. Government and Department of Veteran’s Affairs restrictions relating to participant privacy and consent. Requests to access these datasets should be directed to SD scott.damrauer@pennmedicine.upenn.edu. Code to perform analyses in this article are available from the authors upon request (BV, SD), or from github at https://github.com/Bellomot/Athero_NGWAMA_Multitrait_GWAS.
