# Supplementary material for: Multi-Trait Genome-Wide Association Study of Atherosclerosis Detects Novel Pleiotropic Loci
Source: Front Genet. 2022 Feb 2;12:787545. doi: 10.3389/fgene.2021.787545 (PMC8847690; doi:10.3389/fgene.2021.787545)
Supplement: Supplementary file 2 [file DataSheet3.docx]

**N-GWAMA Method**

The N-GWAMA multi-trait GWAS method uses GWAS summary statistics from the univariate trait GWAS to test if there is an association between each SNP with one or more of the traits tested^1^. N-GWAMA performs this test via a summed weighted Z-score approach to perform this test. In brief, N-GWAMA uses LD-score regression (LDSC) to estimate the heritability, genetic covariance, and sample overlap of the tested traits^2,3^. Then for each SNP, the univariate Z-scores are weighted by their sample size and estimated heritability and added together; that sum is standardized using the estimated variance and covariance of the univariate traits to produce the test statistic Z_k_ (Equation 1). Under the null hypothesis, Z_k_ has a standard normal distribution and thus can easily be used to calculate a p-value for each SNP.

Equation 1:

$$Z_{k}=\frac{\sum_{i=1}^{n} {(w}_{ik}Z_{ik})}{\sqrt{\sum_{i=1}^{n} {(w}_{ik}V)+\sum_{i=1}^{n} \sum_{j=1}^{n} (\sqrt{w_{ik}w_{jk}}C_{i,j})}}$$

**N-GWAMA Multi-trait GWAS Pipeline**

The first step of our pipeline was to filter out SNPs that were not in all the input univariate GWAS summary statistics files and align the alleles of each SNP using MRbase^4^. We next ran LDSC on the univariate GWAS files in order to estimate the heritability, genetic covariance, and sample overlap of the traits^2,3^. Using the MRbase formatted summary statistic files and the LDSC results, we ran N-GWAMA to perform the multi-trait GWAS on each of the SNPs^1^. From the N-GWAMA results, we defined independent loci at the multi-trait genome-wide significant loci using the PLINK command “--clump-r2 0.2”^5^. Finally, we annotated the resulting multi-trait genome-wide significant loci with the genome-wide significant associations within 500KB or r2 > 0.2 from GWAS Catalog. We removed multi-trait genome-wide significant loci from our novel loci list that were annotated with univariate genome-wide significant signal for one or more of the traits involved in the multi-trait GWAS. The code for this pipeline can be found at (<https://github.com/Bellomot/Athero_NGWAMA_Multitrait_GWAS>).

**REFERENCES:**

1. Baselmans, B.M.L., Jansen, R., Ip, H.F., van Dongen, J., Abdellaoui, A., van de Weijer, M.P., Bao, Y., Smart, M., Kumari, M., Willemsen, G., et al. (2019). Multivariate genome-wide analyses of the well-being spectrum. Nat. Genet. *51*, 445–451.

2. Bulik-Sullivan, B.K., Loh, P.-R., Finucane, H.K., Ripke, S., Yang, J., Patterson, N., Daly, M.J., Price, A.L., Neale, B.M., and Neale, B.M. (2015). LD Score regression distinguishes confounding from polygenicity in genome-wide association studies. Nat. Genet. *47*, 291–295.

3. Bulik-Sullivan, B., Finucane, H.K., Anttila, V., Gusev, A., Day, F.R., Loh, P.-R., ReproGen Consortium, R., Psychiatric Genomics Consortium, P.G., Genetic Consortium for Anorexia Nervosa of the Wellcome Trust Case Control Consortium 3, G.C. for A.N. of the W.T.C.C.C., Duncan, L., et al. (2015). An atlas of genetic correlations across human diseases and traits. Nat. Genet. *47*, 1236–1241.

4. Hemani, G., Zheng, J., Elsworth, B., Wade, K.H., Haberland, V., Baird, D., Laurin, C., Burgess, S., Bowden, J., Langdon, R., et al. (2018). The MR-Base platform supports systematic causal inference across the human phenome. Elife *7*,.

5. Purcell, S., Neale, B., Todd-Brown, K., Thomas, L., Ferreira, M.A.R., Bender, D., Maller, J., Sklar, P., De Bakker, P.I.W., Daly, M.J., et al. (2007). PLINK: A tool set for whole-genome association and population-based linkage analyses. Am. J. Hum. Genet. *81*, 559–575.
