# Supplementary material for: Multi-Trait Genome-Wide Association Study of Atherosclerosis Detects Novel Pleiotropic Loci
Source: Front Genet. 2022 Feb 2;12:787545. doi: 10.3389/fgene.2021.787545 (PMC8847690; doi:10.3389/fgene.2021.787545)
Supplement: Supplementary file 3 [file DataSheet1.PDF]

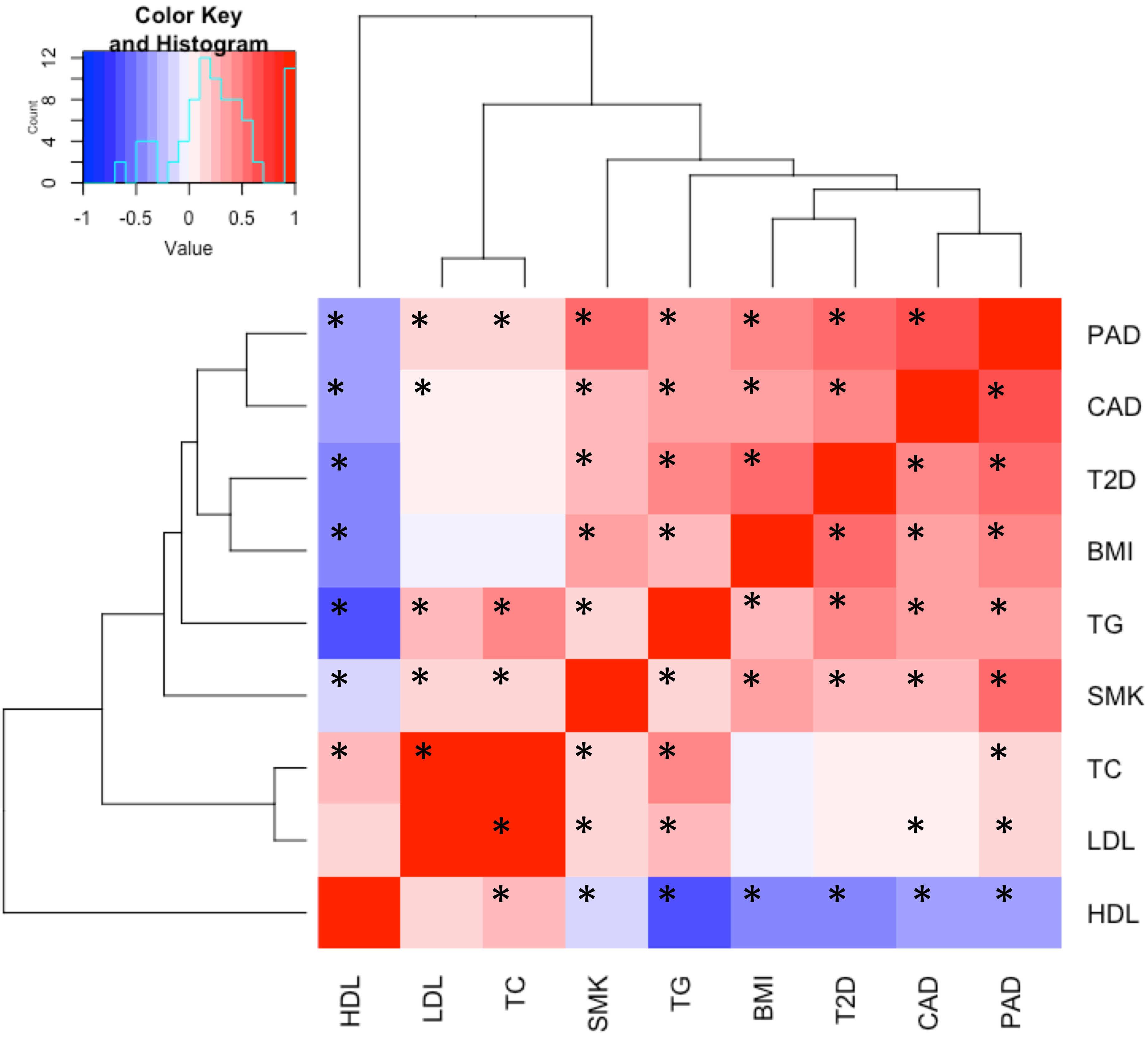

Figure S1. Heat map of 9 traits used in multi-variate analysis. The red represents positive cross-trait LD score regression, or highly positive correlated traits, and the blue represents negative cross-trait LD score regression, or highly negative correlated traits. * in the upper left corner of each box indicates traits were significantly correlated with a p<0.05. Abbreviations: PAD, peripheral artery disease; CAD, coronary artery disease; BMI, body mass index; T2D, type 2 diabetes; SMK, smoking; HDL, high density lipoprotein; LDL, low density lipoprotein; TG, triglycerides; TC, total cholesterol.

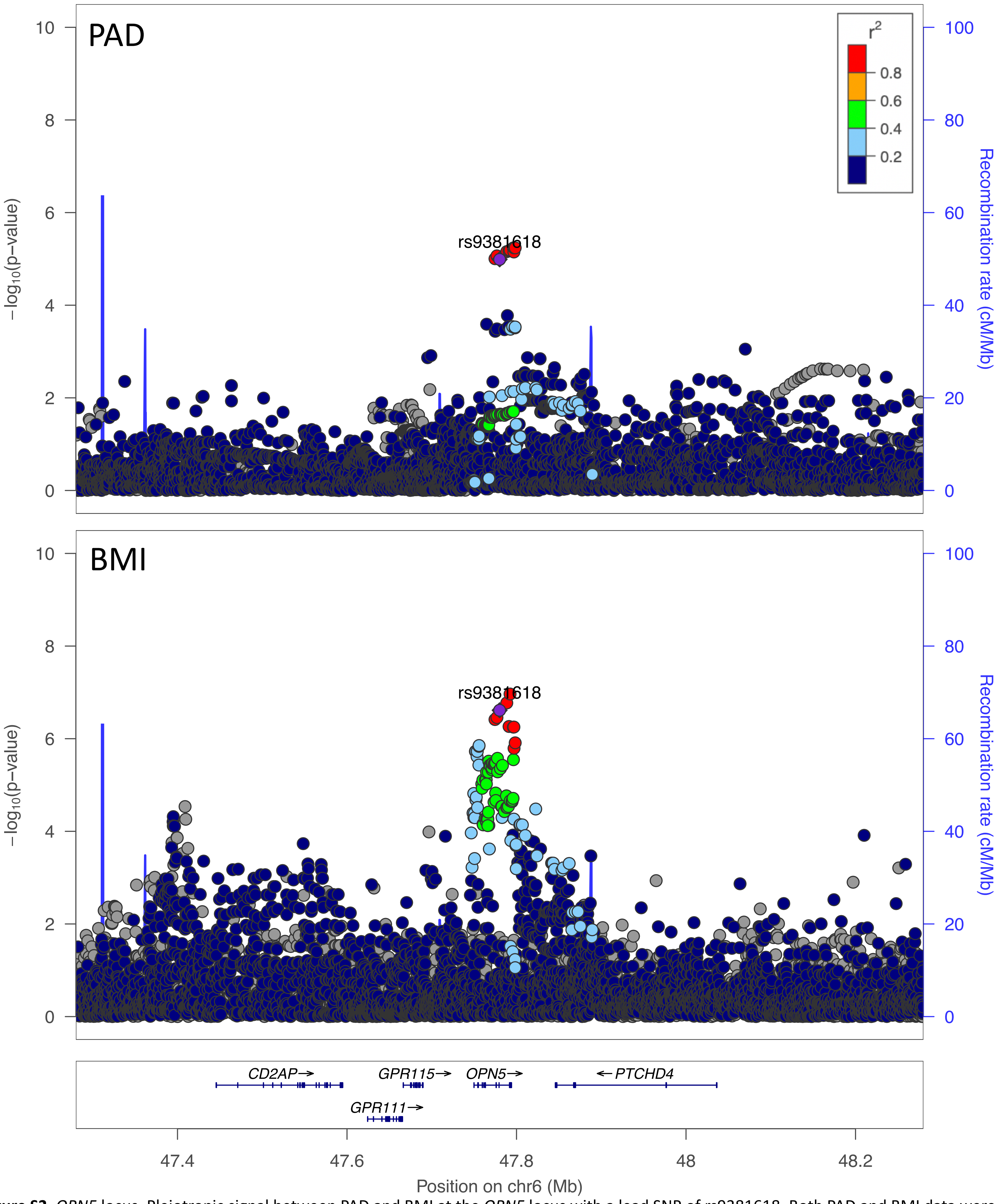

Figure S2. *OPN5* locus. Pleiotropic signal between PAD and BMI at the *OPN5* locus with a lead SNP of rs9381618. Both PAD and BMI data were conditioned on the SNPs rs16876132 and rs7451940 to achieve a conditional posterior probability of colocalization >0.8.

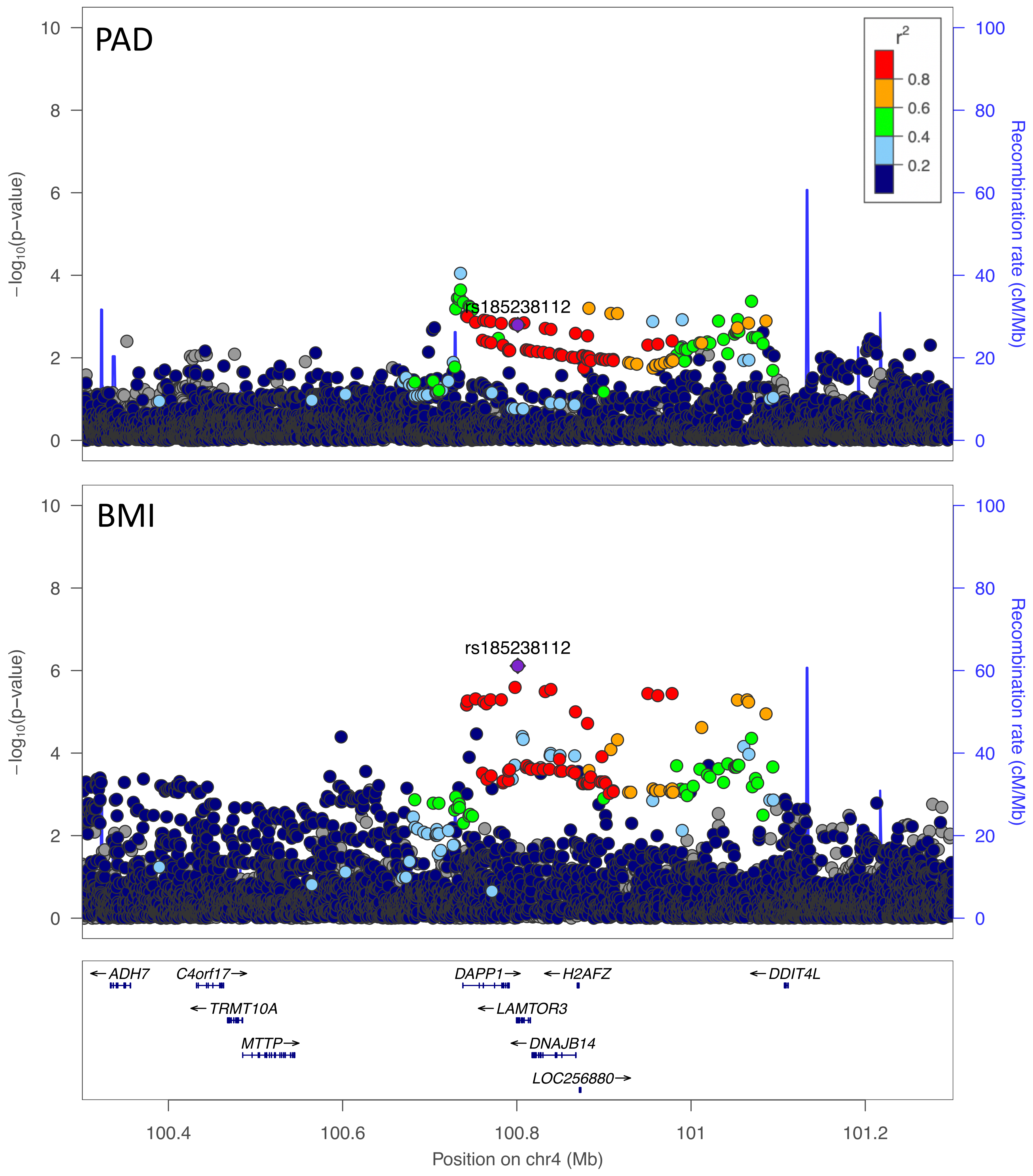

Figure S3. *LTOR3* locus. Pleiotropic signal between PAD and BMI at the *LTOR3* locus with a lead SNP of rs185238112. Both PAD and BMI data were conditioned on the SNP rs62306082 to achieve a conditional posterior probability of colocalization >0.8.

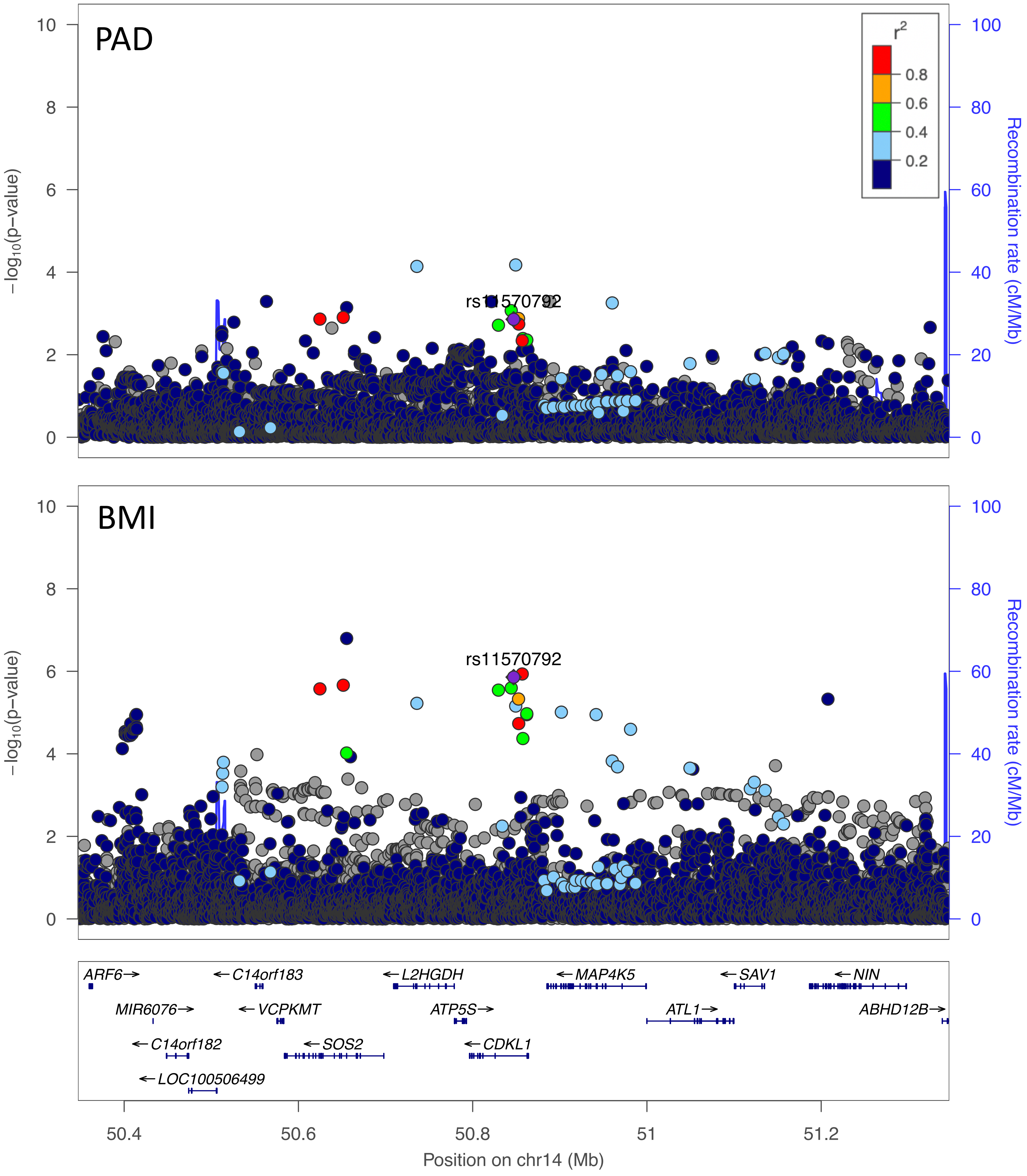

Figure S4. *CDKL1* locus. Pleiotropic signal between PAD and BMI at the *CDKL1* locus with a lead SNP of rs11570792.

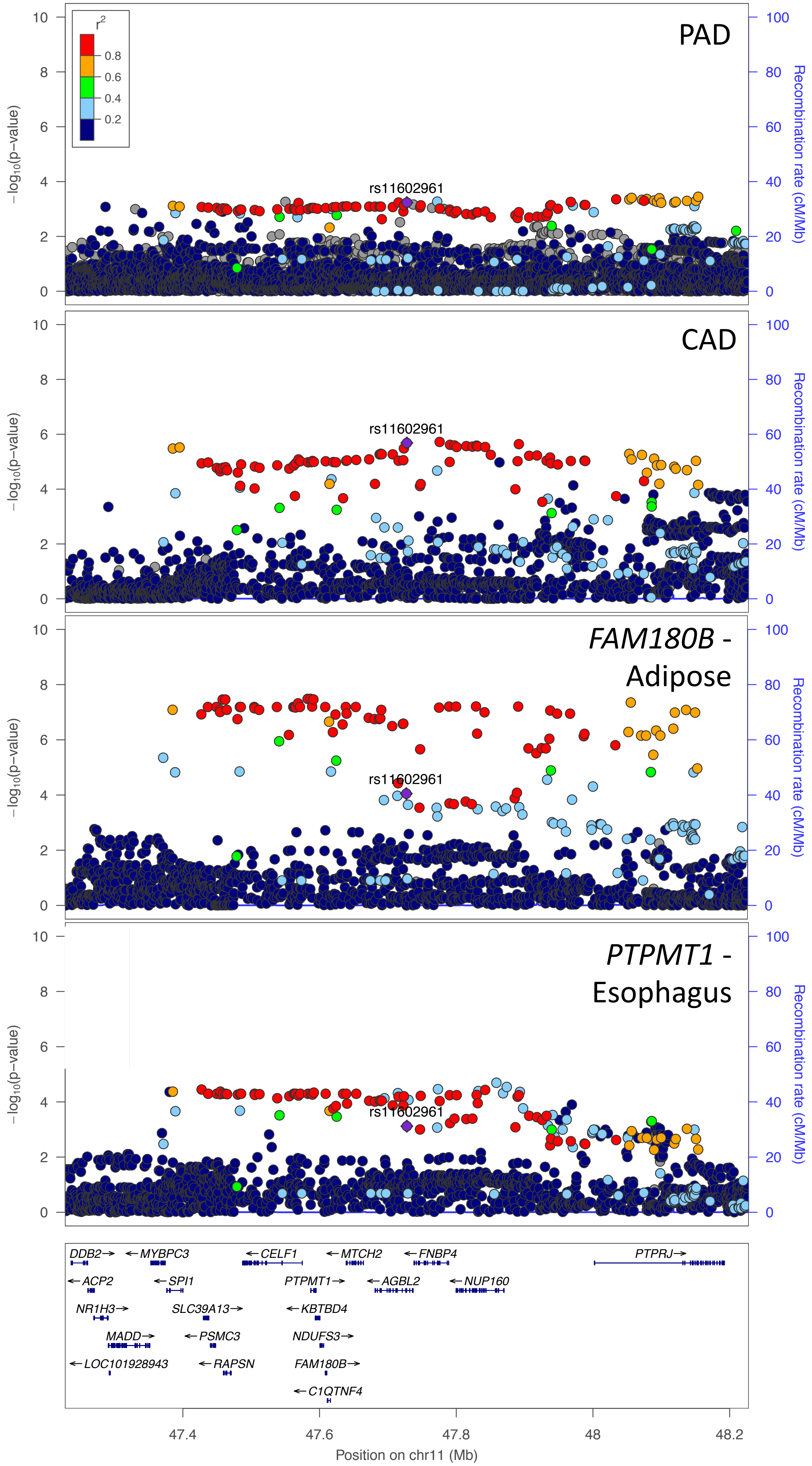

Figure S5. *CBPC2* locus. Pleiotropic signal between PAD and CAD at the *CBPC2* locus with a lead SNP of rs11602961. Both PAD and CAD data were conditioned on the SNP rs116869426 to achieve a conditional posterior probability of colocalization >0.8. The 3rd and 4th panel show the association peak for the eQTLs detected from GTEx v8: *FAM180B* in subcutaneous adipose tissue and *PTPMT1* in esophagus muscularis tissue.

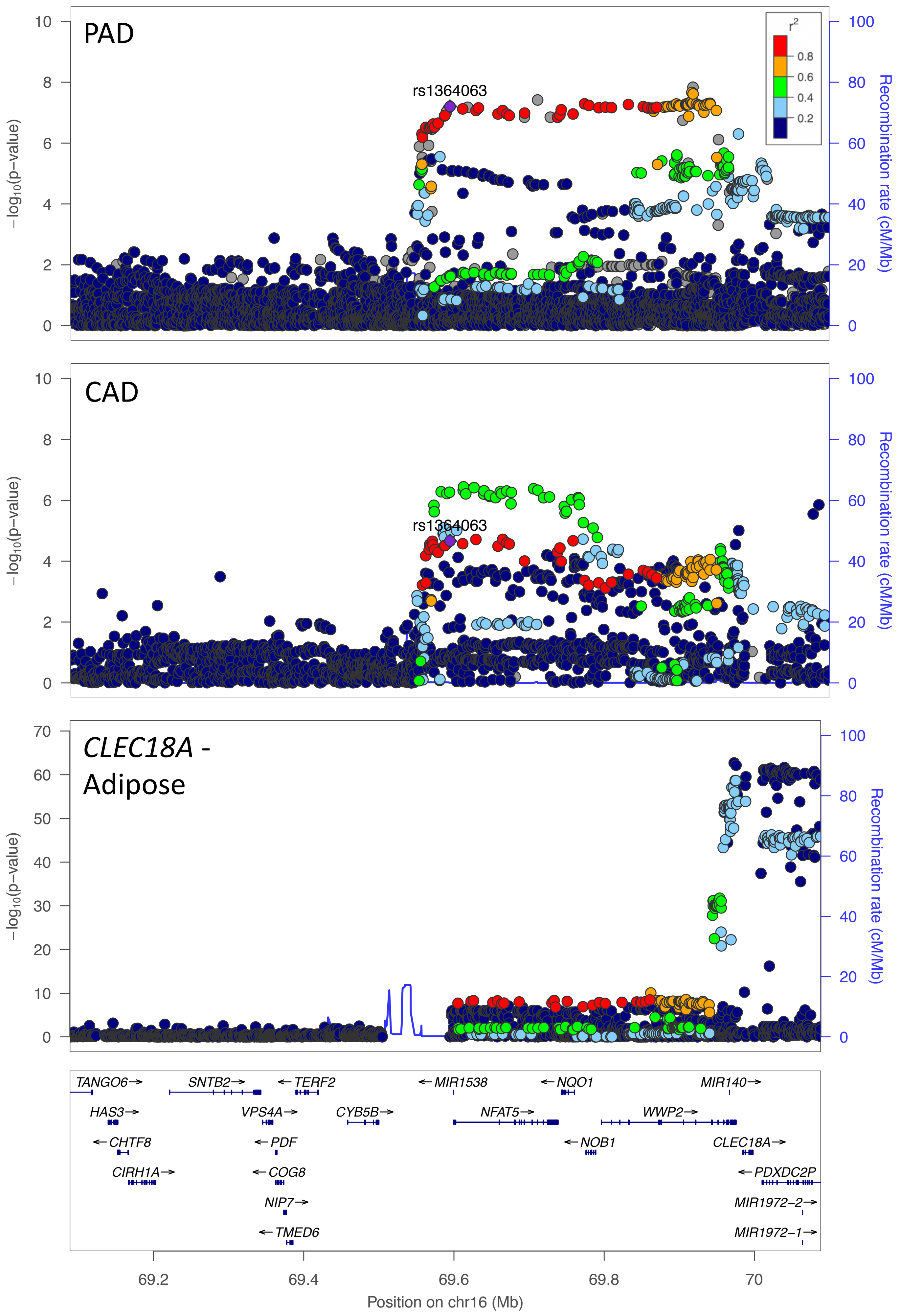

Figure S6. *NFAT5* locus. Pleiotropic signal between PAD and CAD at the *NFAT5* locus with a lead SNP of rs1364063. The 3rd panel shows the association peak of the GTEx v8 eQTL data for the gene *CLEC18A* in adipose subcutaneous tissue.

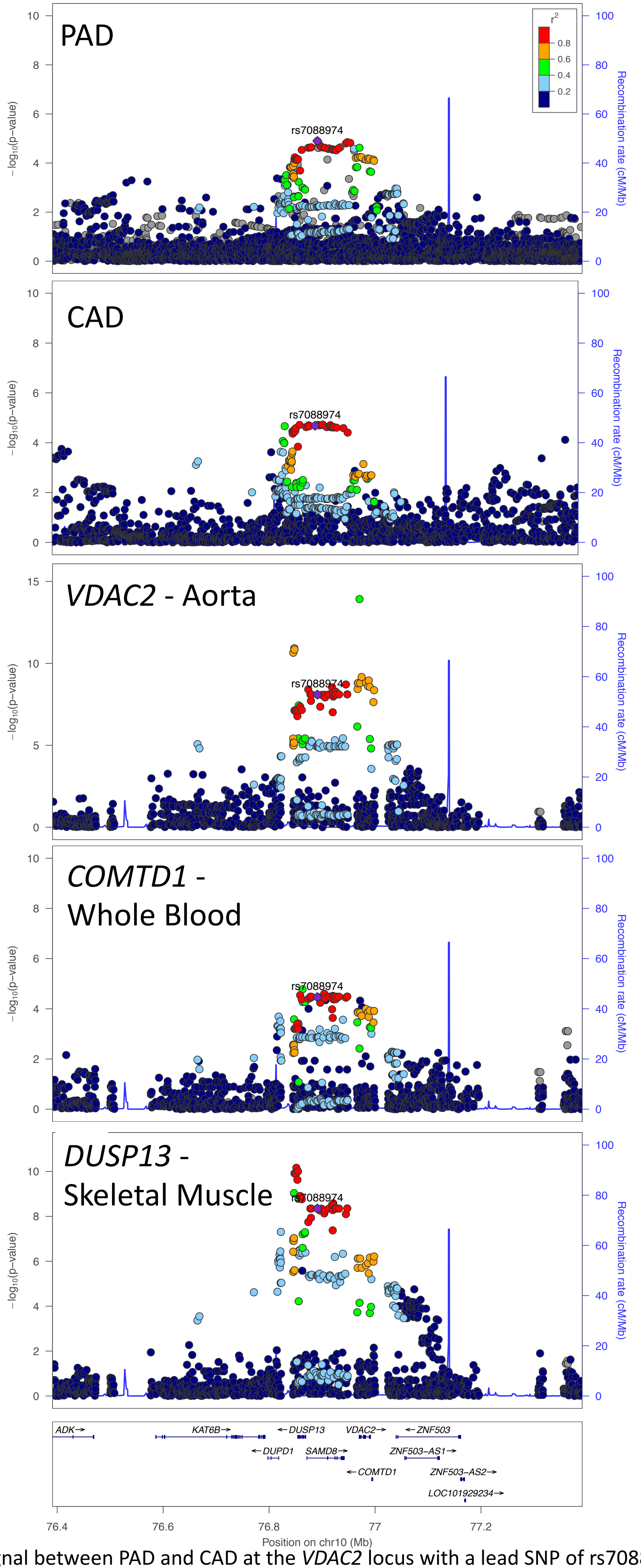

Figure S7. *VDAC2* locus. Pleiotropic signal between PAD and CAD at the *VDAC2* locus with a lead SNP of rs7088974. The bottom 3 panels show the association peak for each eQTL detected from GTEx v8: *VDAC2* in aortic artery, *COMTD1* in whole blood, and *DUSP13* in skeletal muscle tissue.

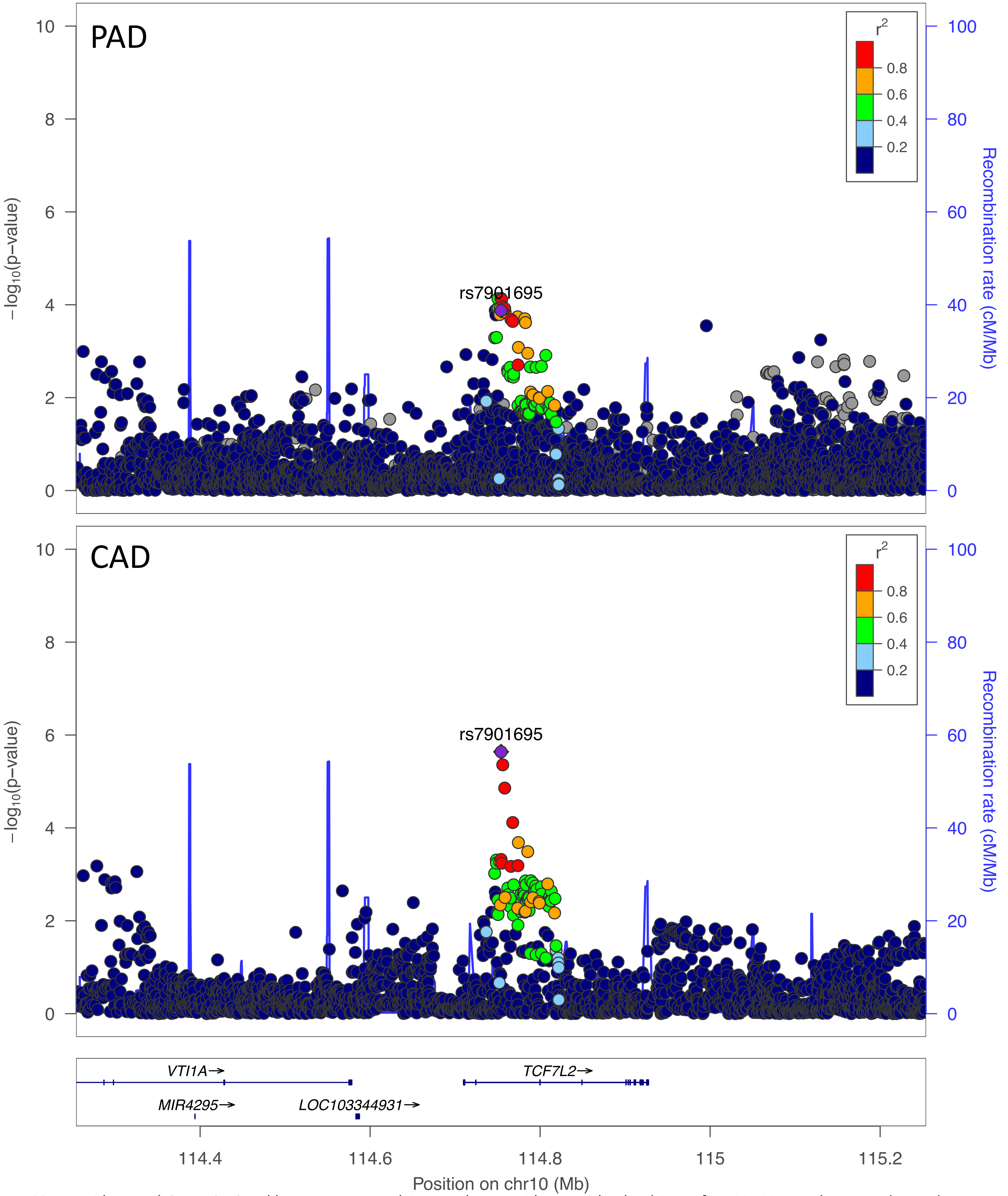

Figure S8. *TF7L2* locus. Pleiotropic signal between PAD and CAD at the *TF7L2* locus with a lead SNP of rs7901695. Both PAD and CAD data were conditioned on the SNP rs57225583 to achieve a conditional posterior probability of colocalization >0.8.

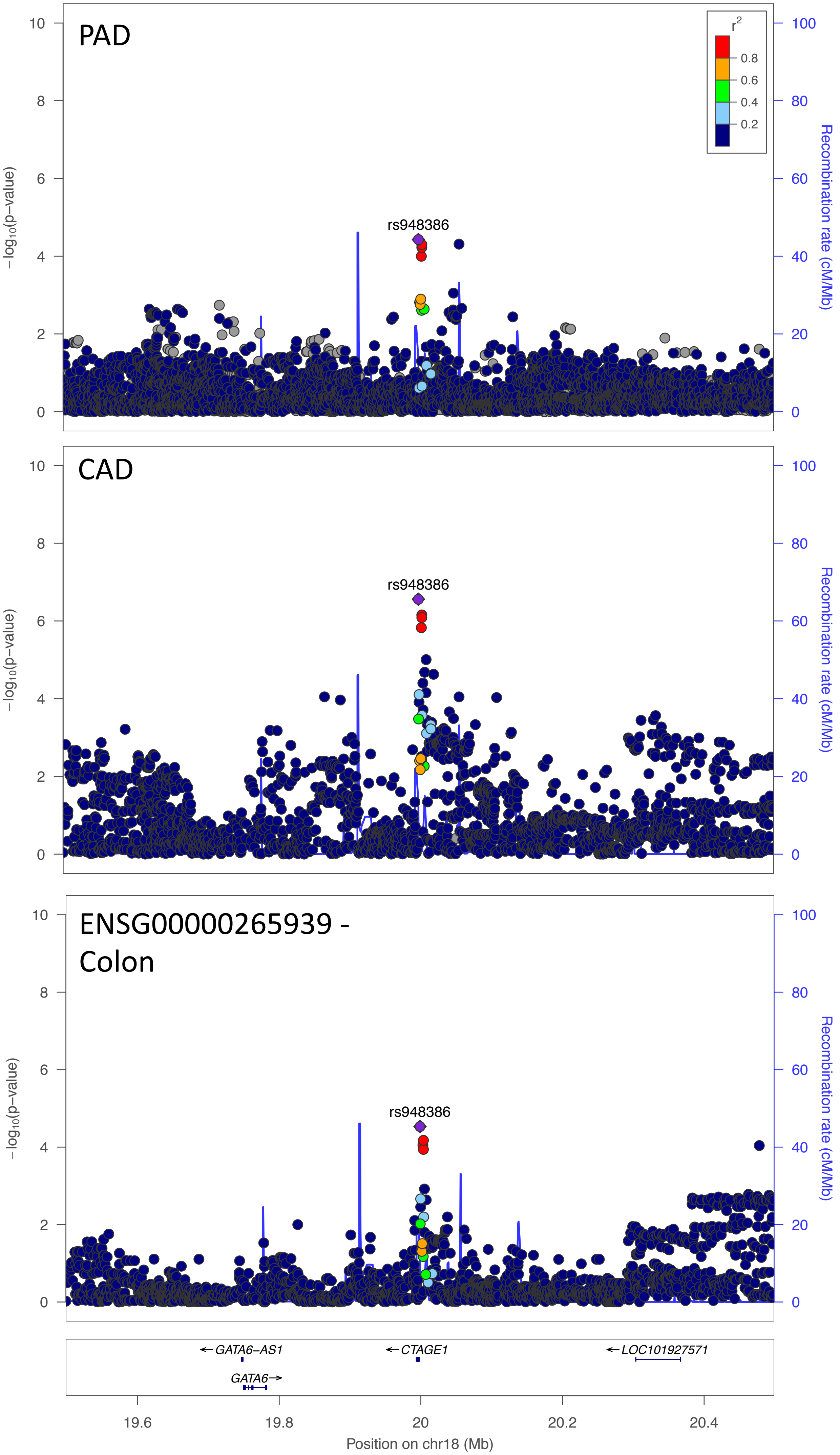

Figure S9. *CTGE1/CTGE2* locus. Pleiotropic signal between PAD and CAD at the *CTGE1/CTGE2* locus with a lead SNP of rs948386. Both PAD and CAD data were conditioned on the SNPs rs112091464 and rs6507183 to achieve a conditional posterior probability of colocalization >0.8. The 3rd panel shows the association peak of the GTEx v8 eQTL data for the gene ENSG00000265939 in transverse colon tissue.

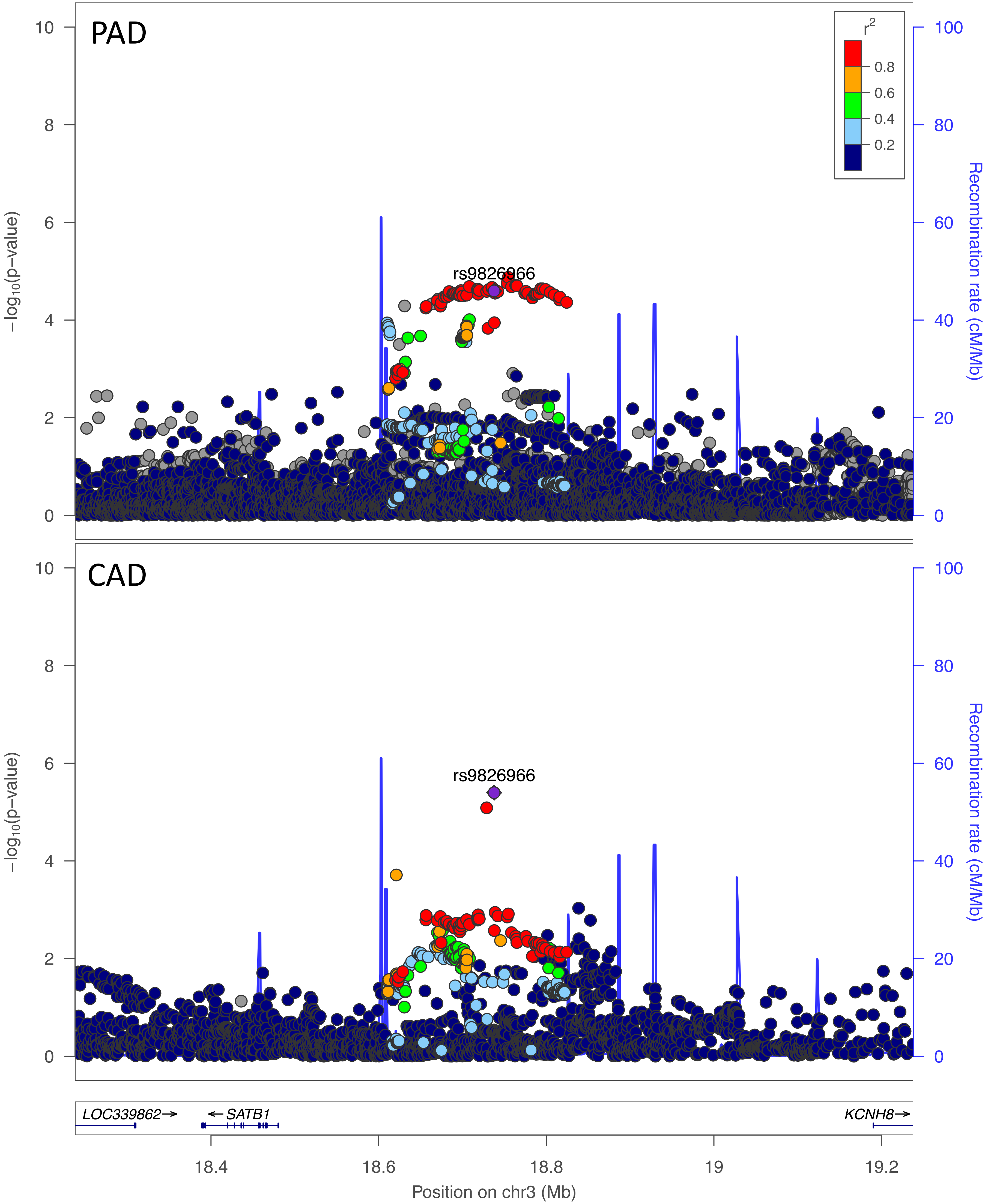

Figure S10. *SATB1* locus. Pleiotropic signal between PAD and CAD at the *SATB1* locus with a lead SNP of rs9826966.

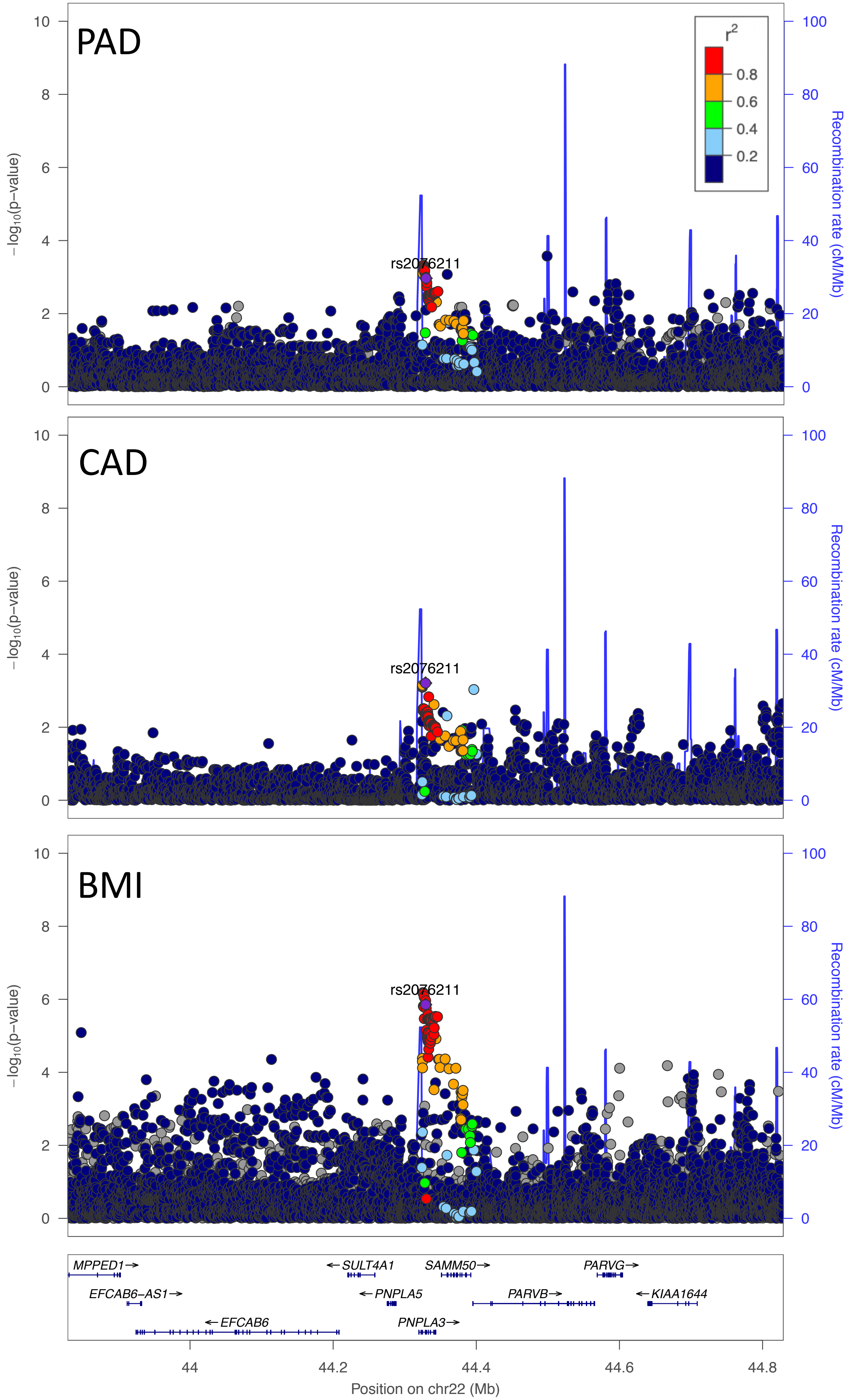

Figure S11. *PNPLA3* locus. Pleiotropic signal between PAD, CAD, and BMI at the *PNPLA3* locus with a lead SNP of rs2076211. PAD, CAD, and BMI data were conditioned on the SNPs rs34123977, rs2179559, and rs71330735 to achieve a conditional posterior probability of colocalization >0.8.

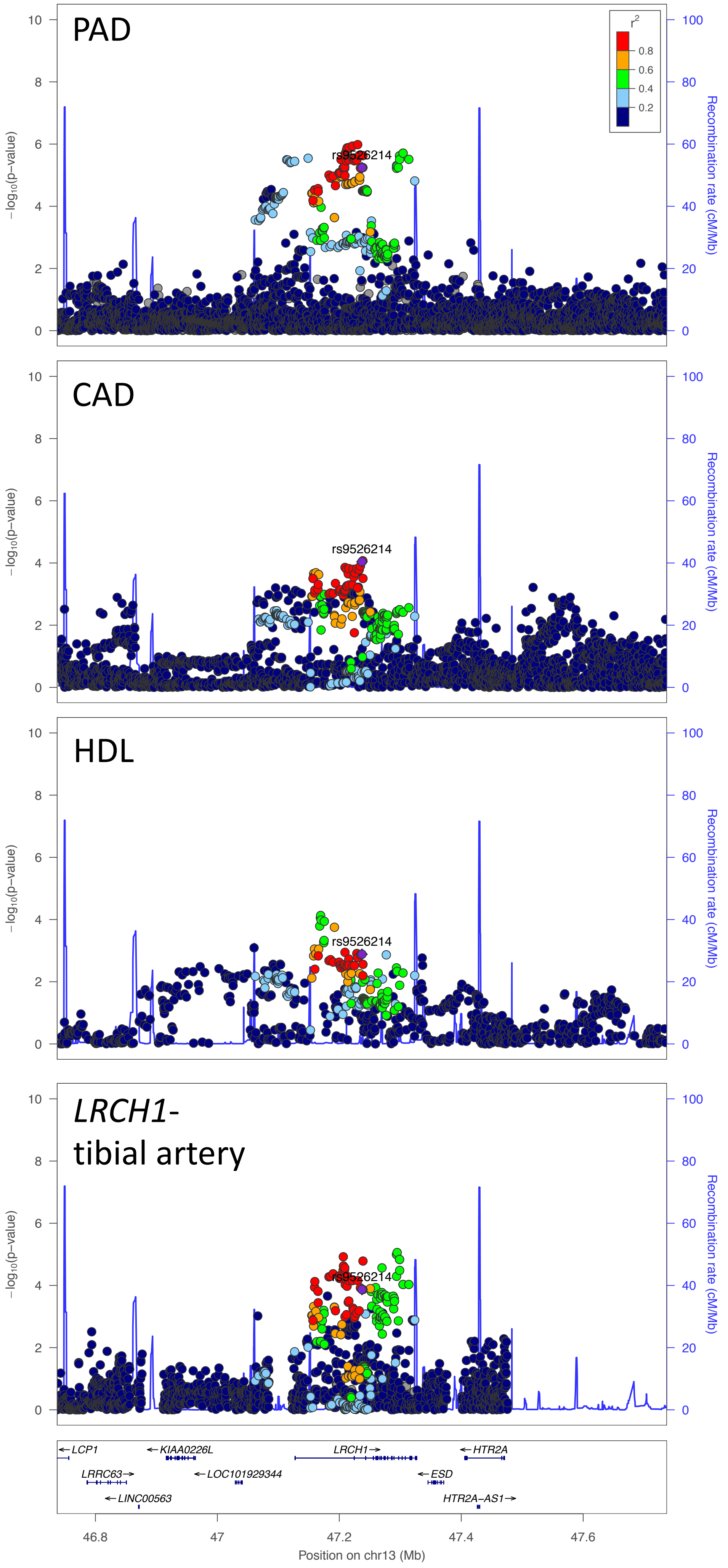

Figure S12. *LRCH1* locus. Pleiotropic signal between PAD, CAD, and HDL at the *LRCH1* locus with a lead SNP of rs9526214. PAD, CAD, and HDL data were conditioned on the SNP rs9316223 to achieve a conditional posterior probability of colocalization >0.8. The 3rd panel shows the association peak of the GTEx v8 eQTL data for the gene *LRCH1* in tibial artery tissue.

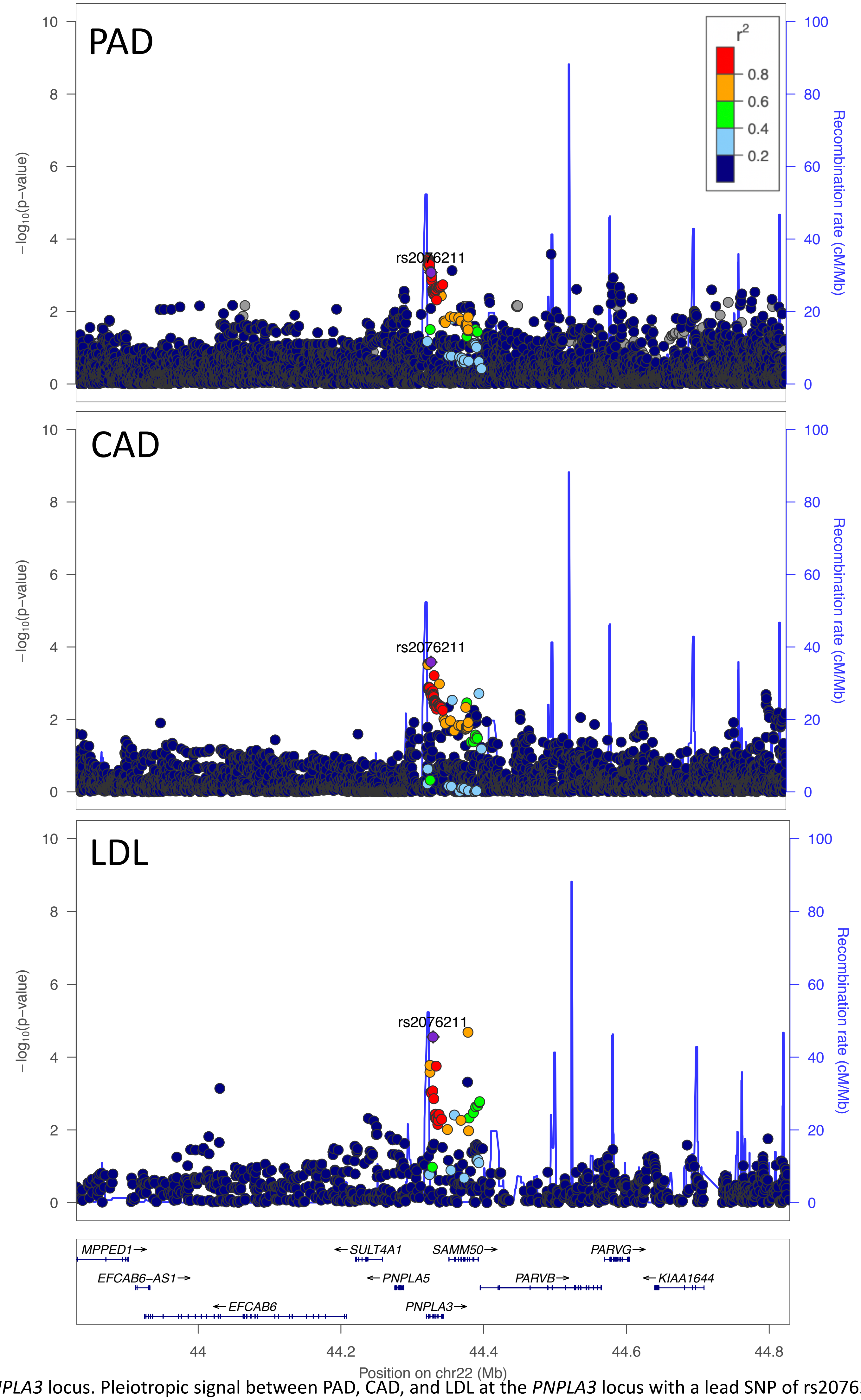

Figure S13. *PNPLA3* locus. Pleiotropic signal between PAD, CAD, and LDL at the *PNPLA3* locus with a lead SNP of rs2076211. PAD, CAD, and LDL data were conditioned on the SNPs rs34123977 and rs9614207 to achieve a conditional posterior probability of colocalization >0.8. This locus was also detected in the PAD, CAD, and BMI scan.

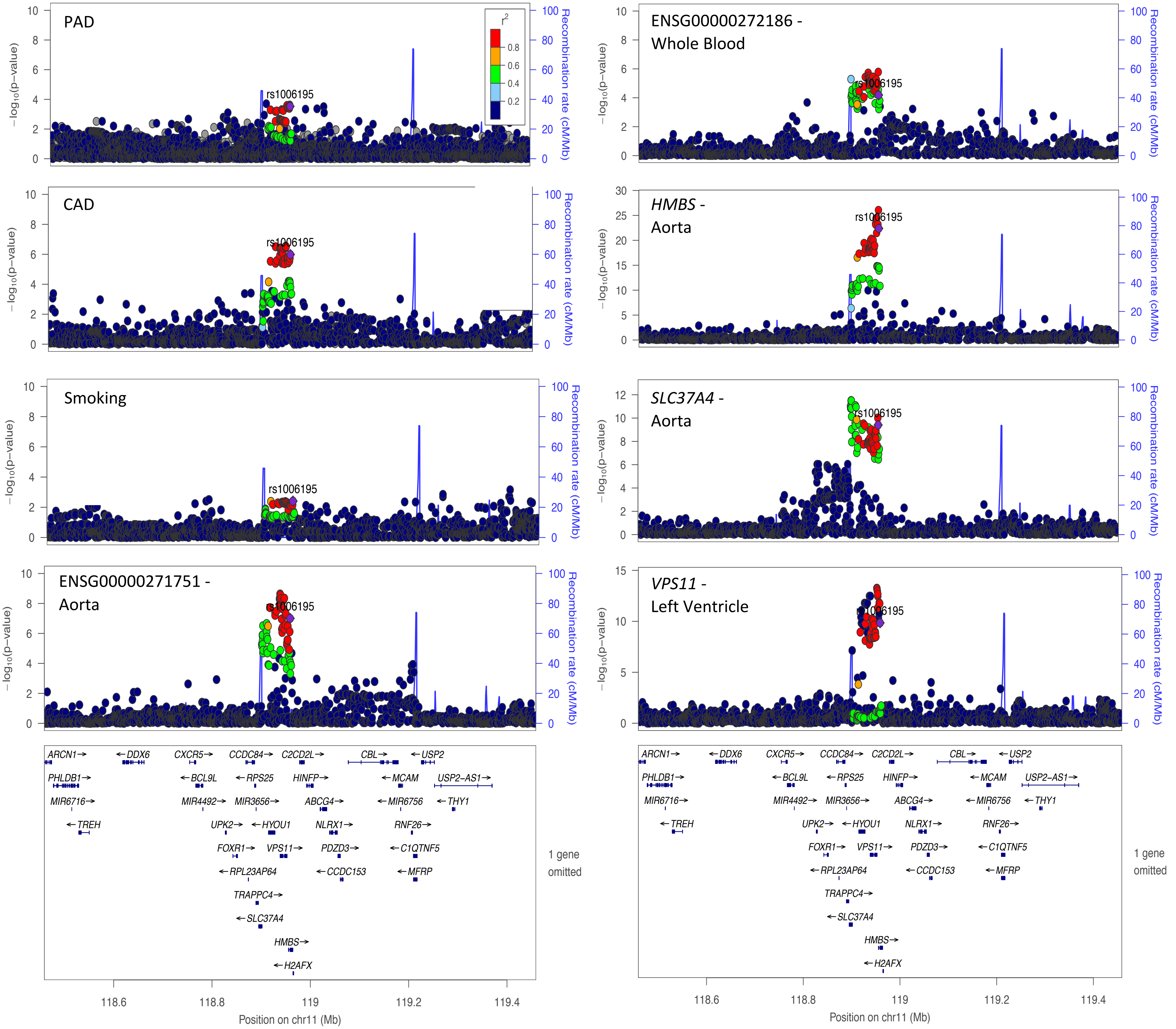

Figure S14. *HMBS* locus. Pleiotropic signal between PAD, CAD, and SMK at the *HMBS* locus with a lead SNP of rs1006195. The bottom left panel and the 4 panels on the right show the association peak for each gene eQTL detected from GTEx v8: ENSG00000271751 in aortic artery, ENSG00000272186 in whole blood, *HMBS* in aortic artery, *SLC37A4* in aortic artery, and VPS11 in the heart left ventricle tissue.

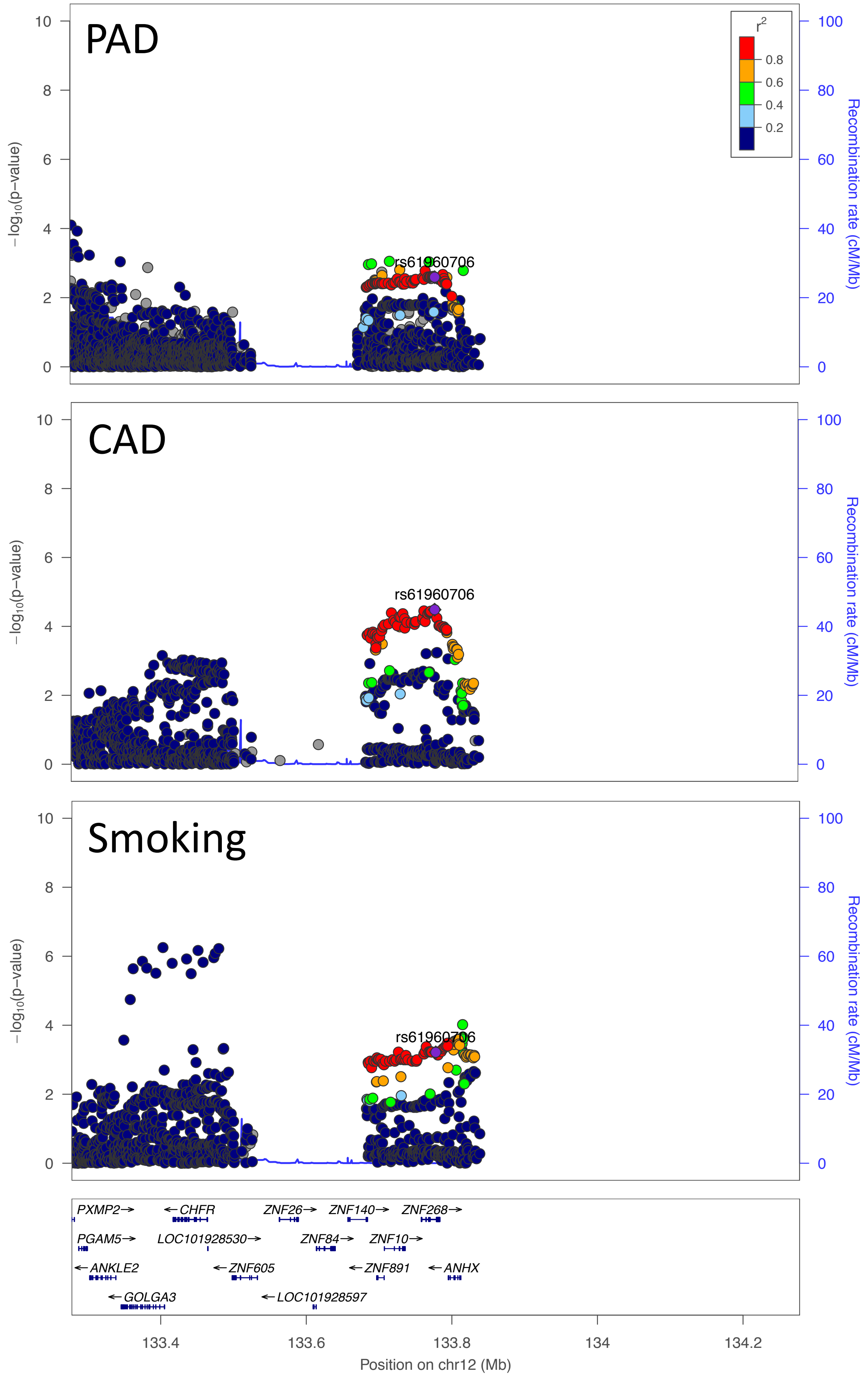

Figure S15. *ZN268* locus. Pleiotropic signal between PAD, CAD, and SMK at the *ZN268* locus with a lead SNP of rs61960706.

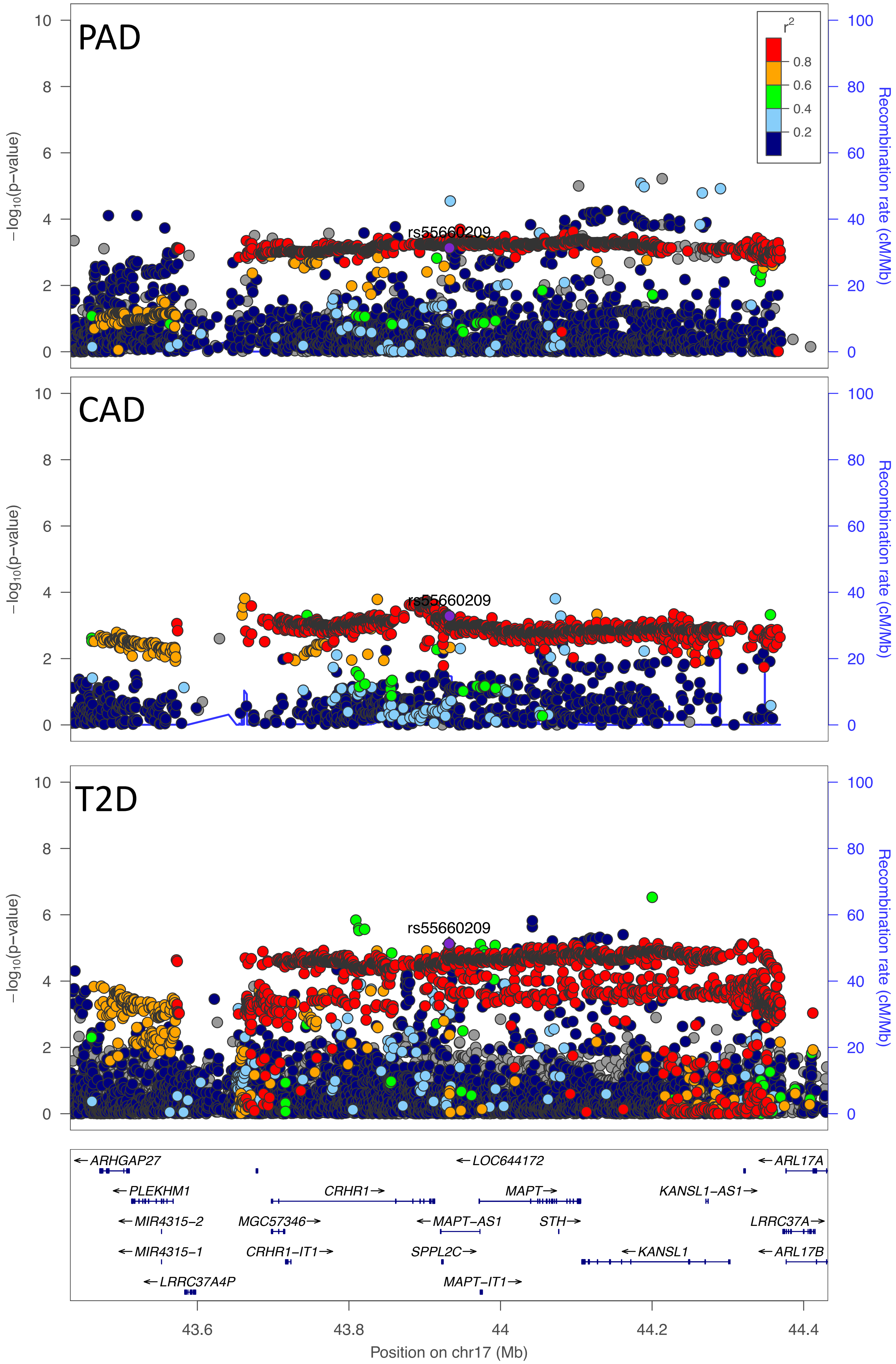

Figure S16. *SPP2C* locus. Pleiotropic signal between PAD, CAD, and T2D at the SPP2C locus with a lead SNP of rs55660209.

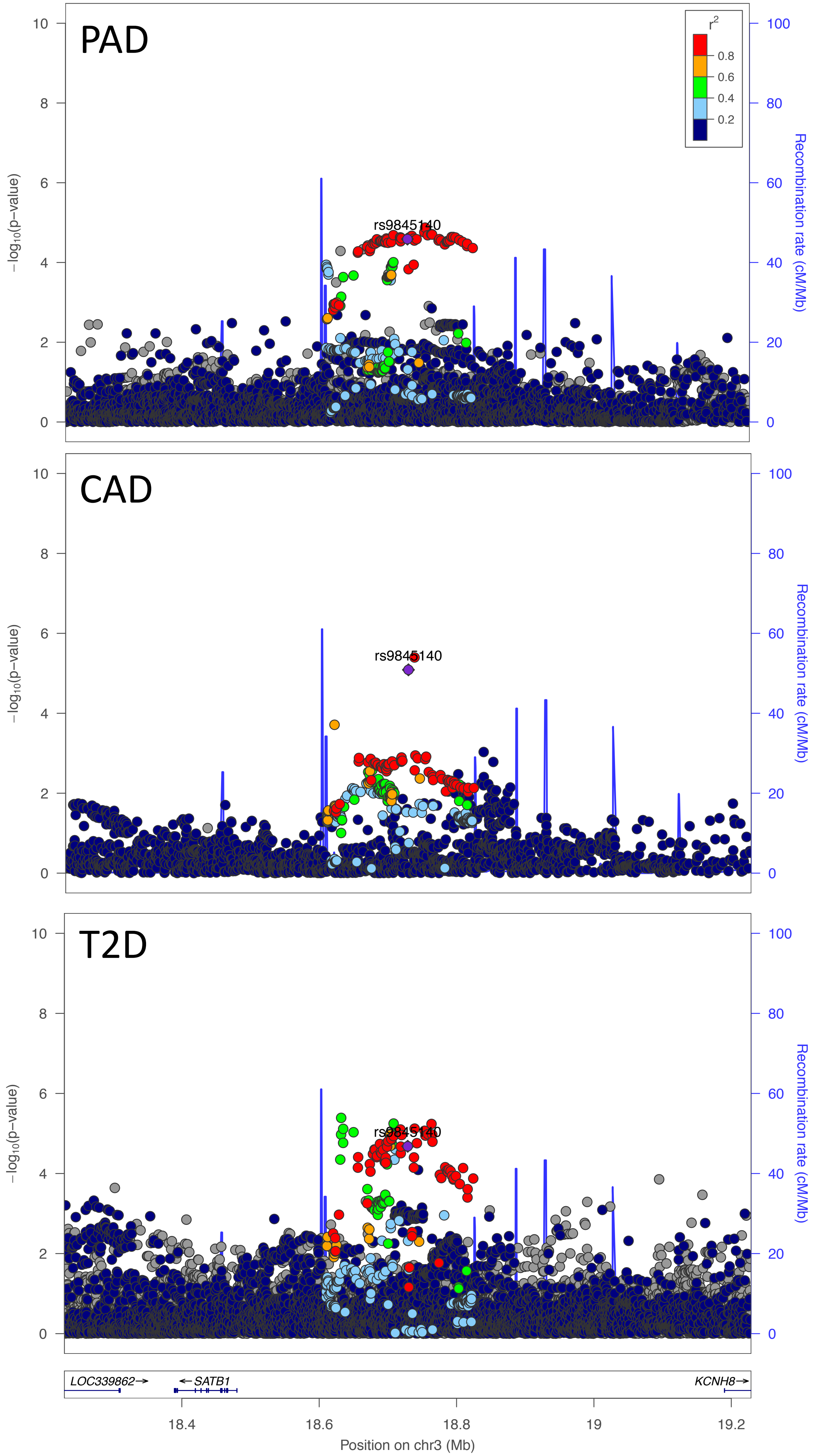

Figure S17. *SATB1* locus. Pleiotropic signal between PAD, CAD, and T2D at the *SATB1* locus with a lead SNP of rs9845140.

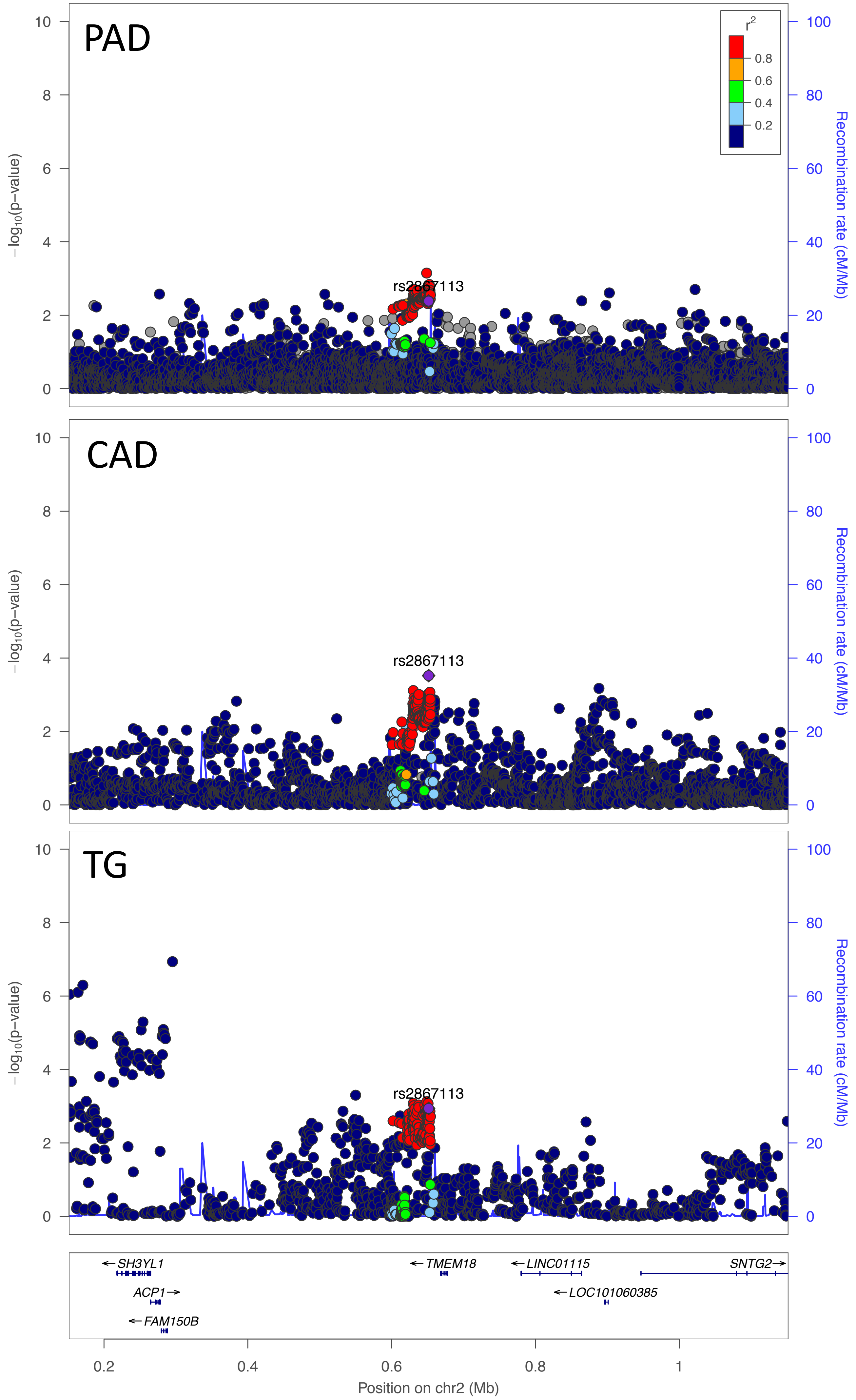

Figure S18. *TMM18* locus. Pleiotropic signal between PAD, CAD, and TG at the *TMM18* locus with a lead SNP of rs2867113. PAD, CAD, and TG data were conditioned on the SNPs rs10180960, rs2685263, rs6728929, rs142804281, and rs11683176 to achieve a conditional posterior probability of colocalization >0.8.

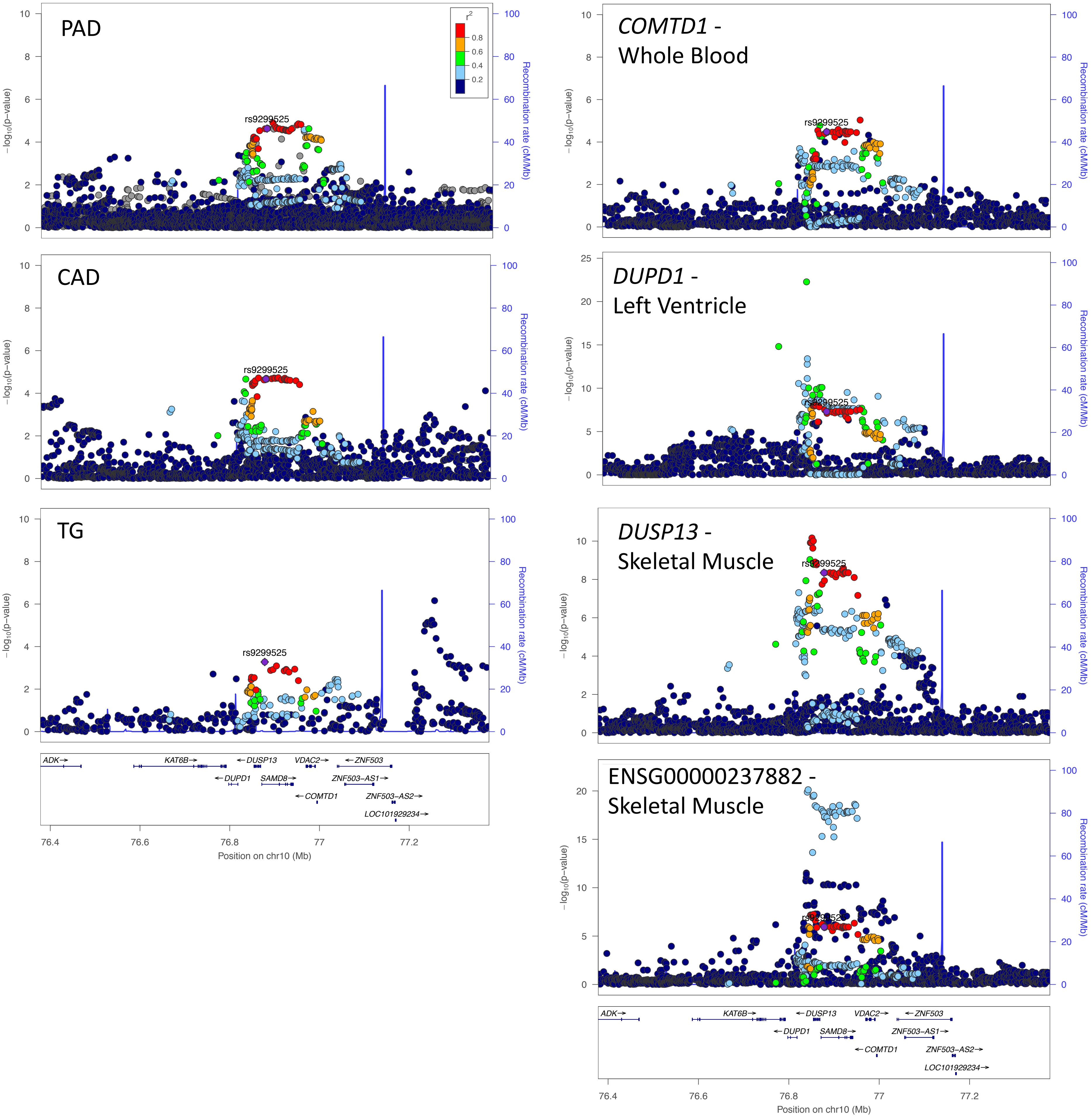

Figure S19. *SAMD8* locus. Pleiotropic signal between PAD, CAD, and TG at the *SAMD8* locus with a lead SNP of rs9299525. The 4 panels on the right show the association peak for each gene eQTL detected from GTEx v8: *COMTD1* in whole blood, *DUPD1* in heart left ventricle tissue, *DUSP13* in skeletal muscle tissue, and ENSG00000237882 in skeletal muscle tissue.

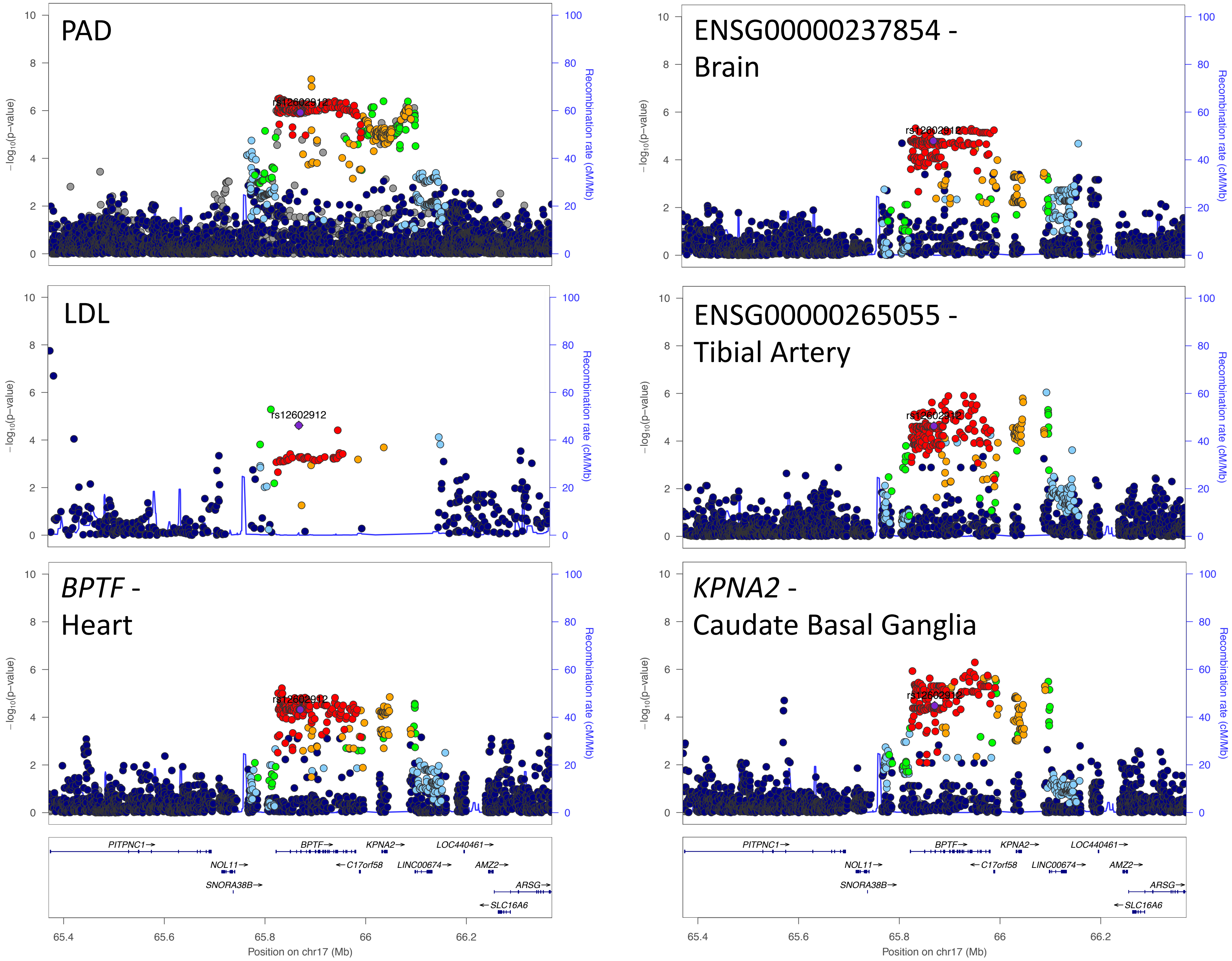

Figure S20. *BPTF* locus. Pleiotropic signal between PAD and LDL at the *BPTF* locus with a lead SNP of rs12602912. Both PAD and LDL data were conditioned on the SNP rs7224860 to achieve a conditional posterior probability of colocalization >0.8. The bottom left and 3 right panels show the association peak for each gene eQTL detected from GTEx v8: *BPTF* in heart atrial appendage tissue, ENSG00000237854 in brain frontal cortex BA, ENSG00000265055 in tibial artery tissue, and KPNA2 in the caudate basal ganglia.

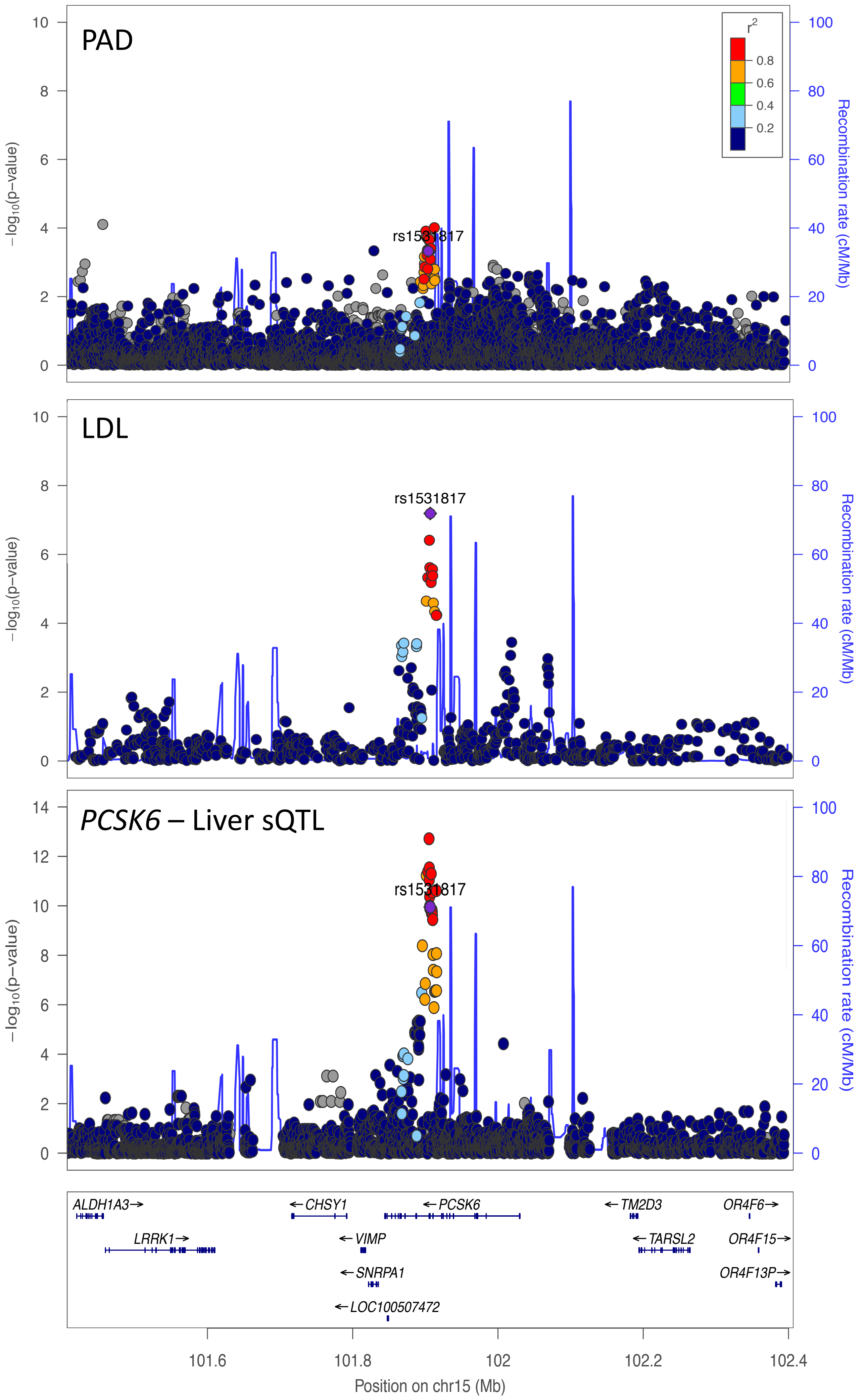

Figure S21. *PCSK6* locus. Pleiotropic signal between PAD and LDL at the *PCSK6* locus with a lead SNP of rs1531817. The 3rd panel shows the association peak of sQTL data for the gene *PCSK6* in liver tissue.

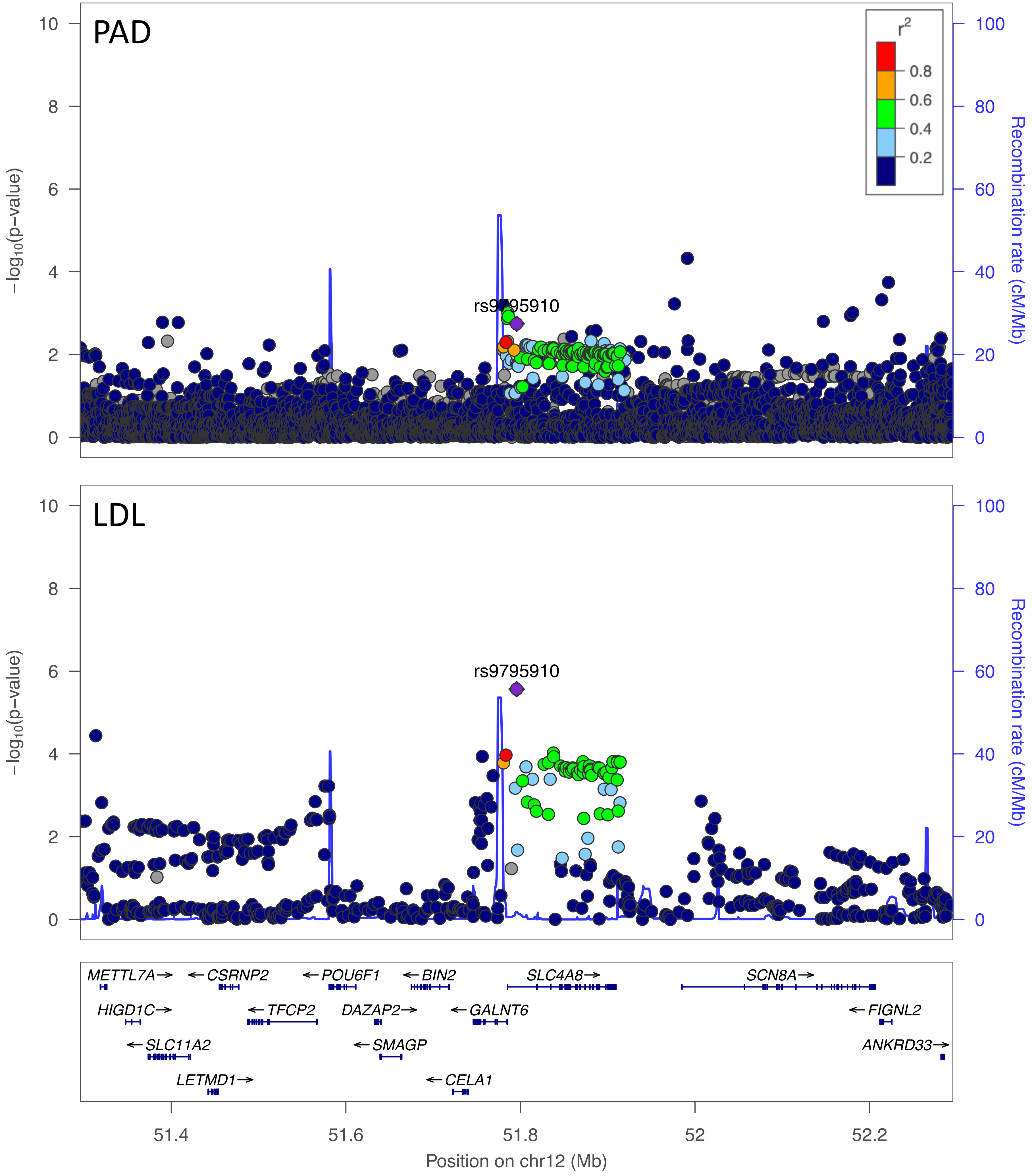

Figure S22. *S4A8* locus. Pleiotropic signal between PAD and LDL at the *S4A8* locus with a lead SNP of rs9795910. This locus was also detected in the PAD TC scan.

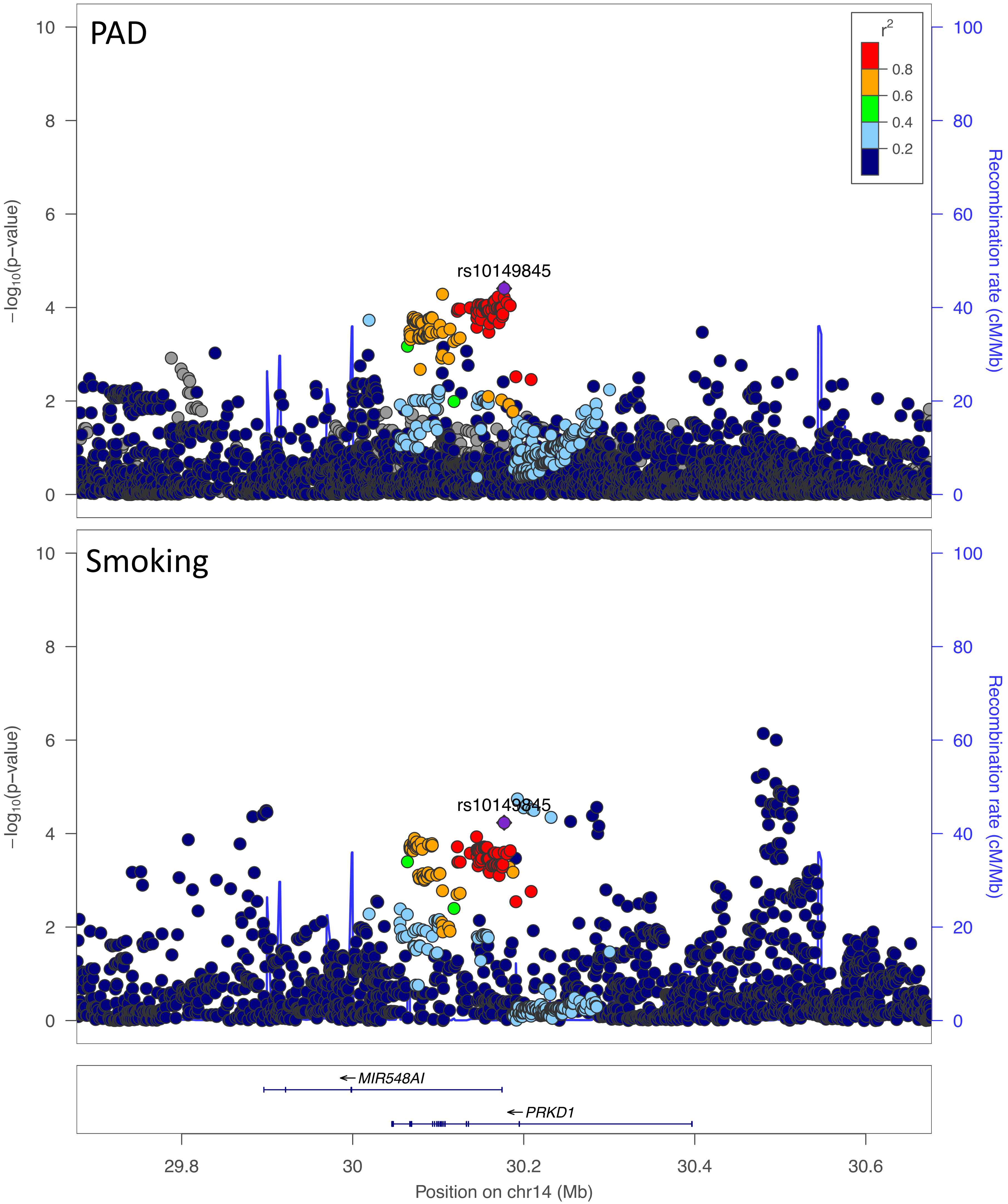

Figure S23. *KPCD1* locus. Pleiotropic signal between PAD and Smoking at the KPCD1 locus with a lead SNP of rs10149845. PAD and Smoking data were conditioned on the SNP rs118049178 to achieve a conditional posterior probability of colocalization >0.8.

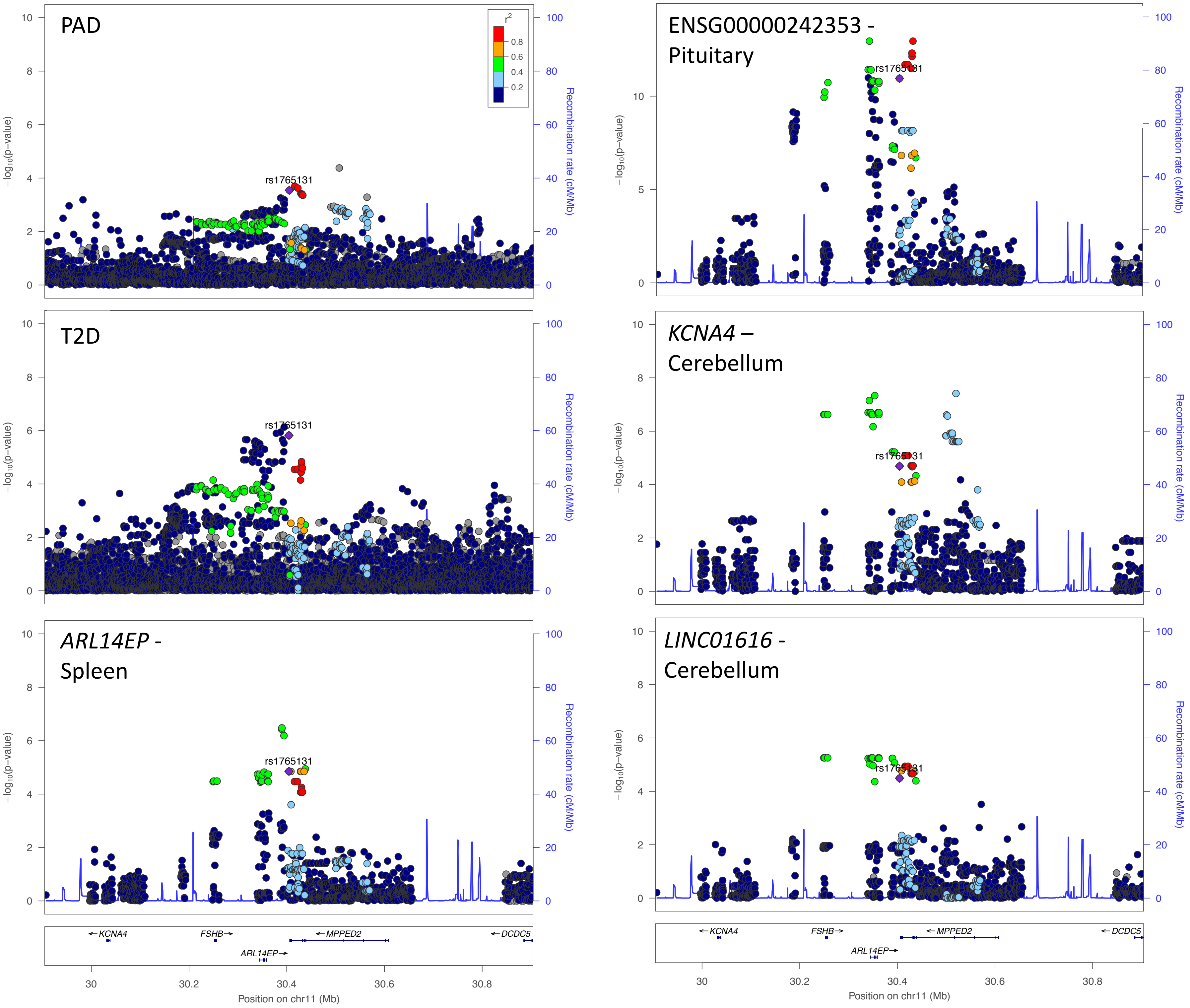

Figure S24. *MPPD2* locus. Pleiotropic signal between PAD and T2D at the *MPPD2* locus with a lead SNP of rs1765131. Both PAD and T2D data were conditioned on the SNPs rs7125524, rs10835690, and rs78132205 to achieve a conditional posterior probability of colocalization >0.8. The bottom left and 3 right panels show the association peak for each gene eQTL detected from GTEx v8: *ARL14EP* in Spleen tissue, ENSG00000242353 in pituitary, *KCNA4* in brain cerebellum tissue, and *LINC01616* in brain cerebellum tissue.

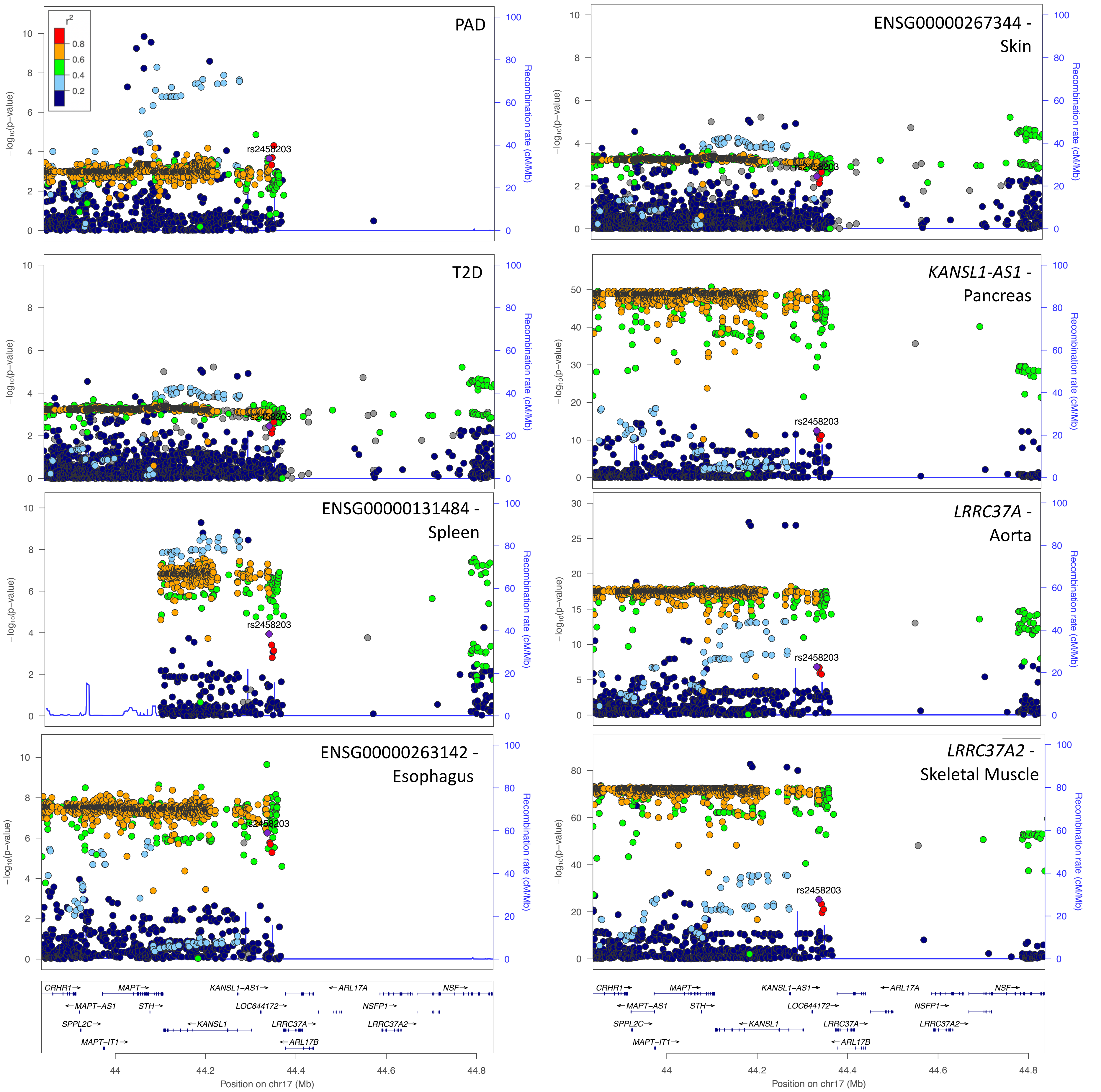

Figure S25. *ARL17* locus. Pleiotropic signal between PAD and T2D at the *ARL17* locus with a lead SNP of rs2458203. The 2 bottom left and 4 right panels show the association peak for each gene eQTL detected from GTEx v8: ENSG00000131484 in spleen tissue, ENSG00000263142 in esophagus mucosa, ENSG00000267344 in suprapubic skin not exposed to sun, *KANSL1-AS1* in pancreas tissue, *LRRC37A* in aortic artery, and *LRRC37A2* in skeletal muscle.

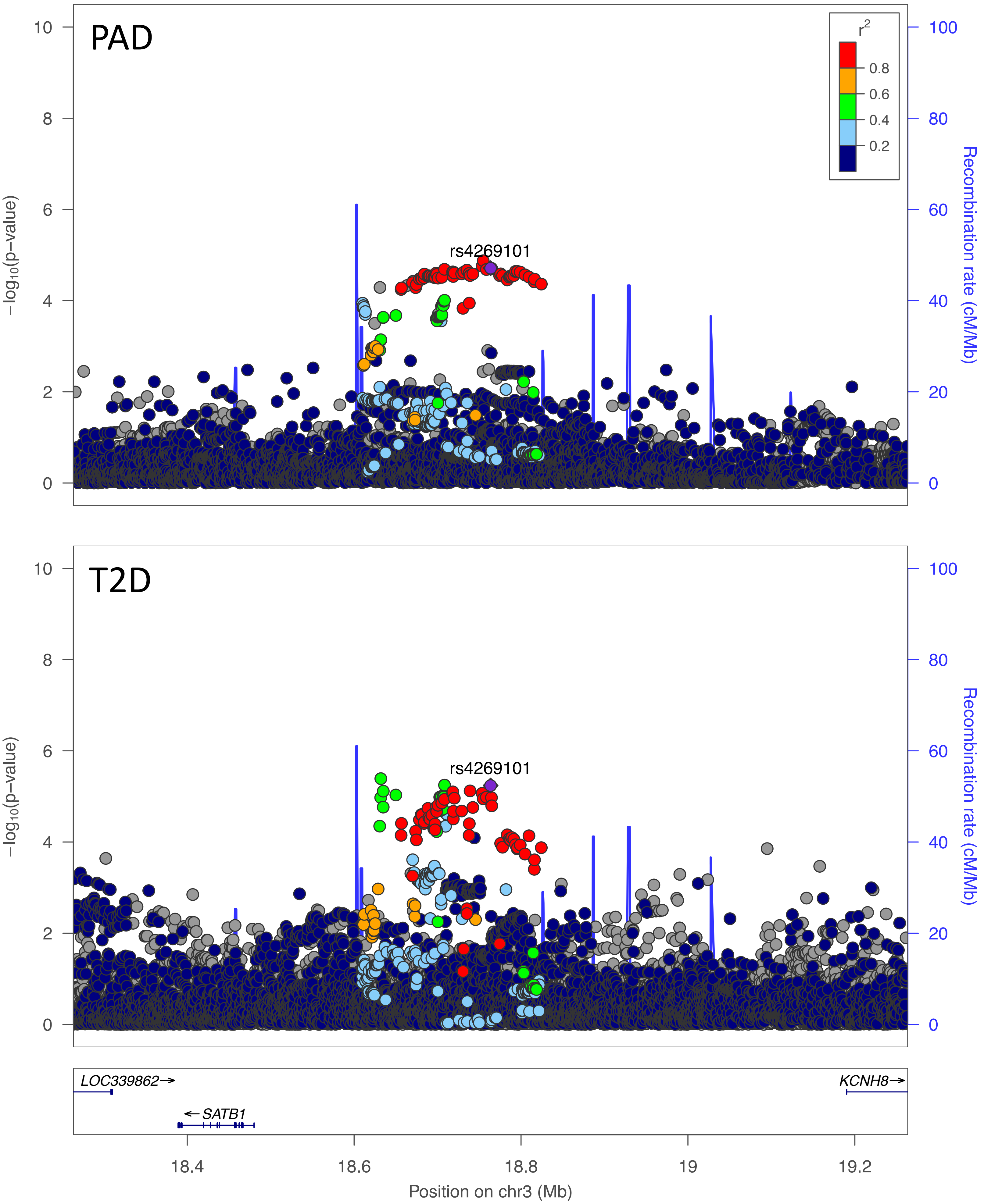

Figure S26. *SATB1* locus. Pleiotropic signal between PAD and T2D at the *SATB1* locus with a lead SNP of rs4269101.

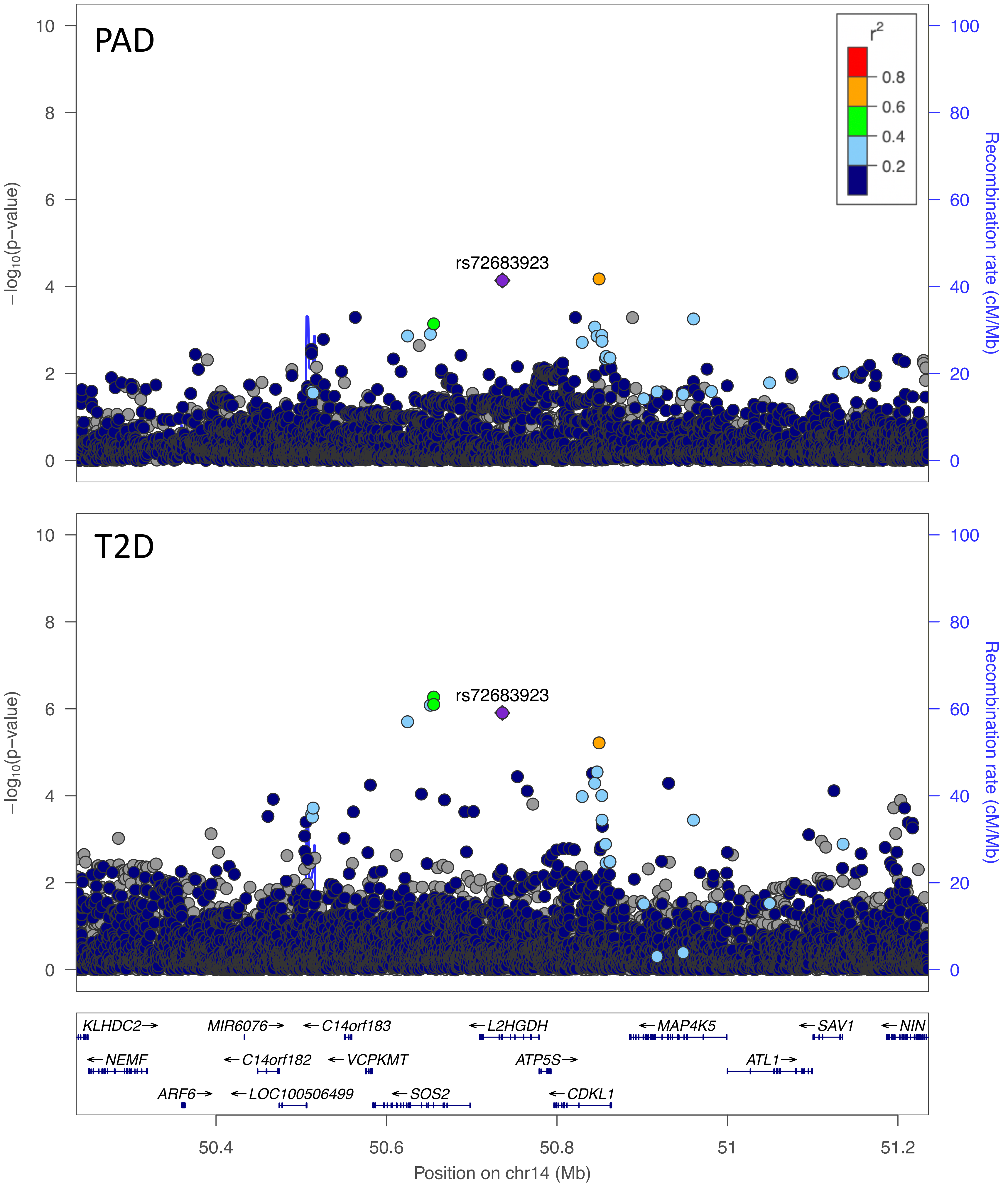

Figure S27. *L2HDH* locus. Pleiotropic signal between PAD and T2D at the *L2HDH* locus with a lead SNP of rs72683923.

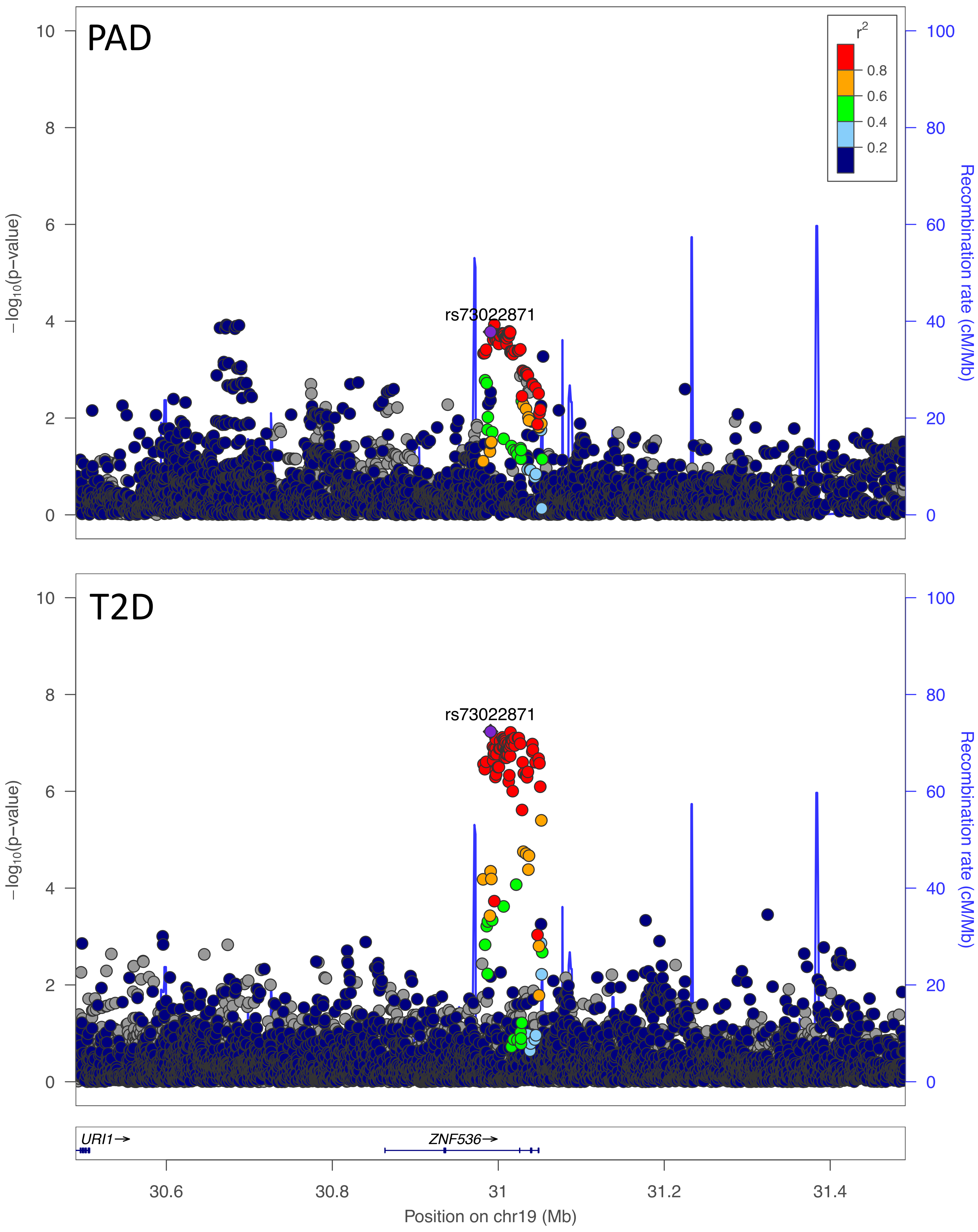

Figure S28. *ZN536* locus. Pleiotropic signal between PAD and T2D at the *ZN536* locus with a lead SNP of rs73022871. Both PAD and T2D data were conditioned on the SNPs rs138985581 and rs17628099 to achieve a conditional posterior probability of colocalization >0.8.

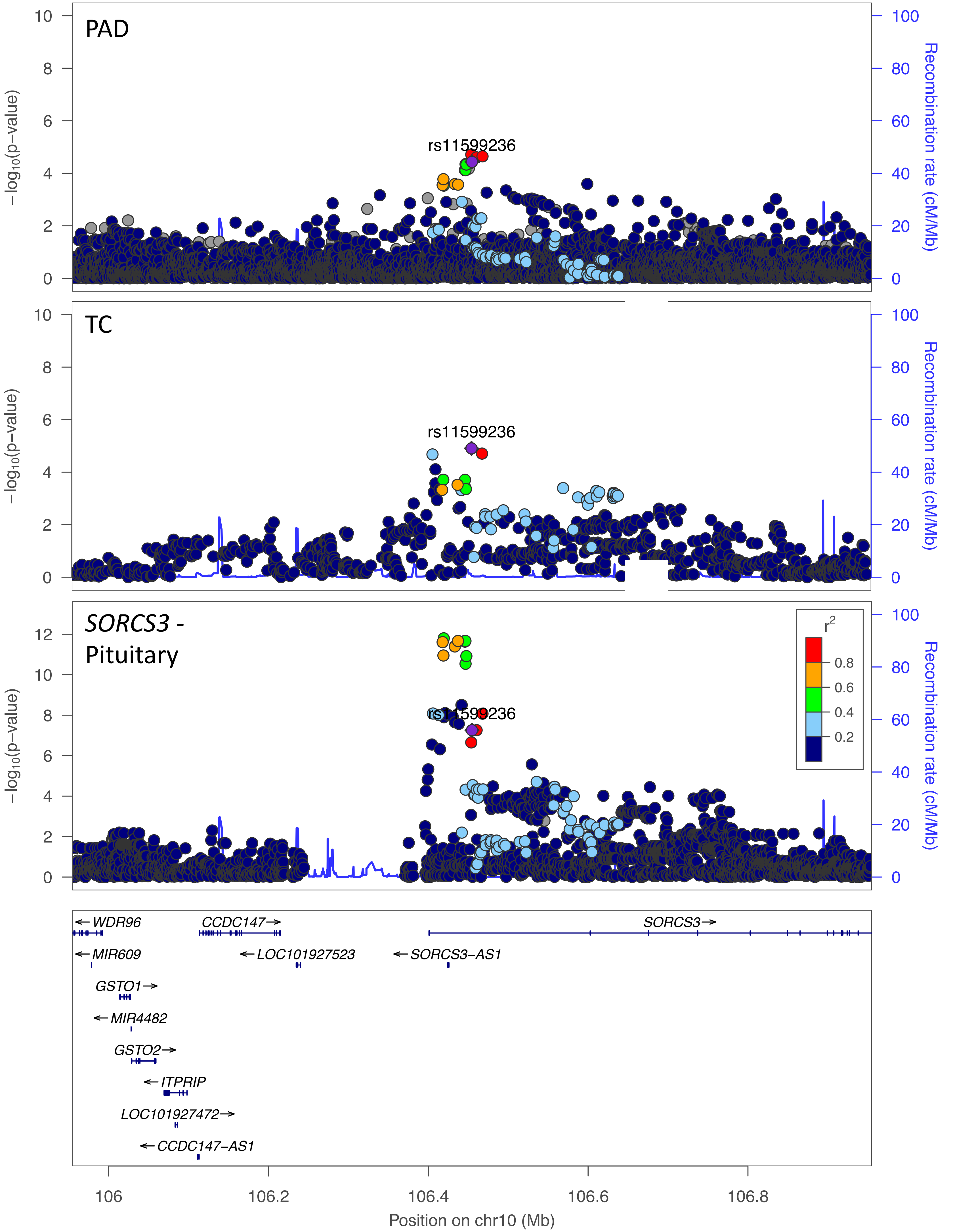

Figure S29. *SORCS3* locus. Pleiotropic signal between PAD and TC at the *SORCS3* locus with a lead SNP of rs11599236. The 3rd panel shows the association peak of eQTL GTEx v8 data for the gene *SORC3* in pituitary tissue.

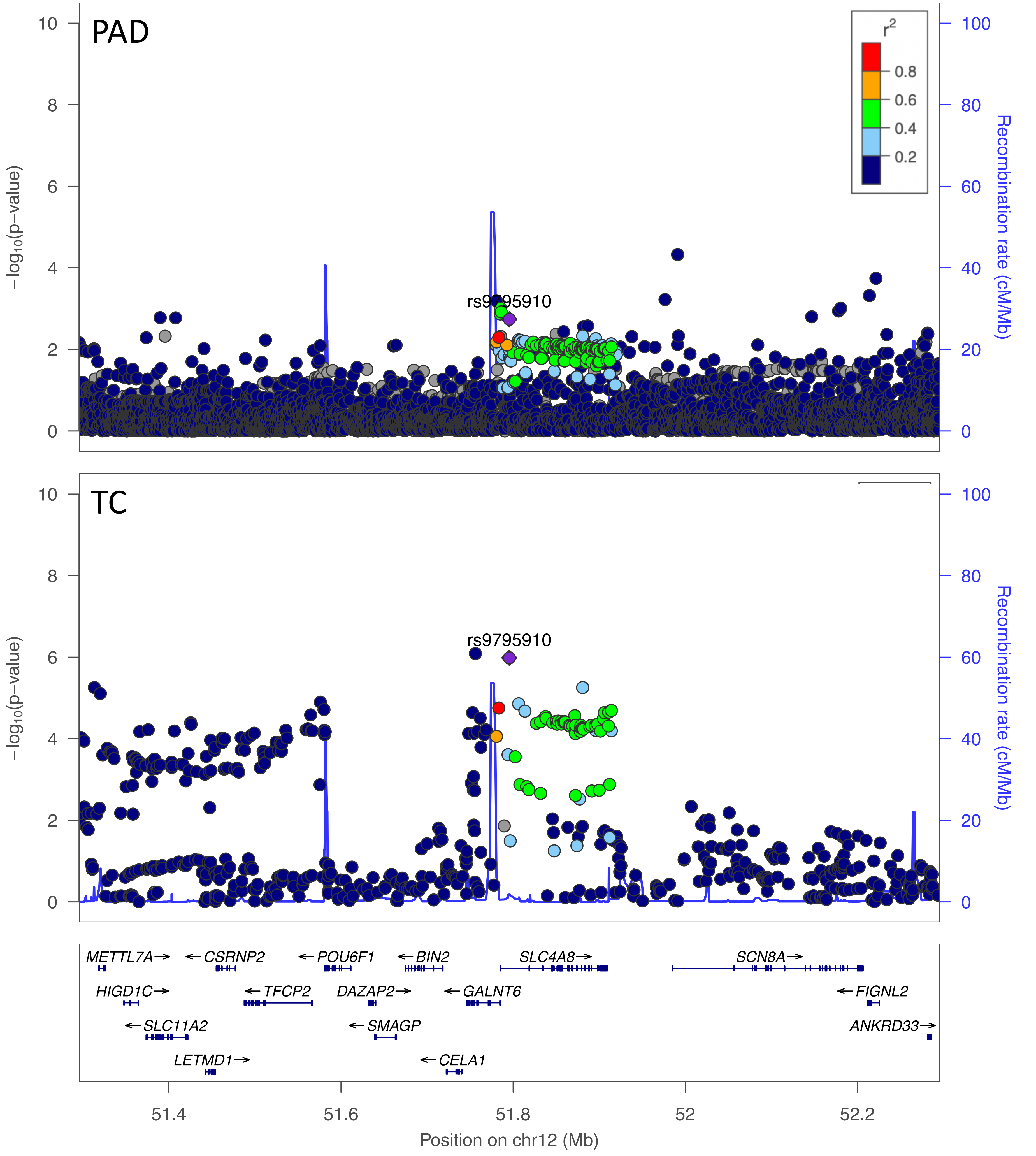

Figure S30. *S4A8* locus. Pleiotropic signal between PAD and TC at the *S4A8* locus with a lead SNP of rs9795910. This locus was also detected in the PAD LDL scan.

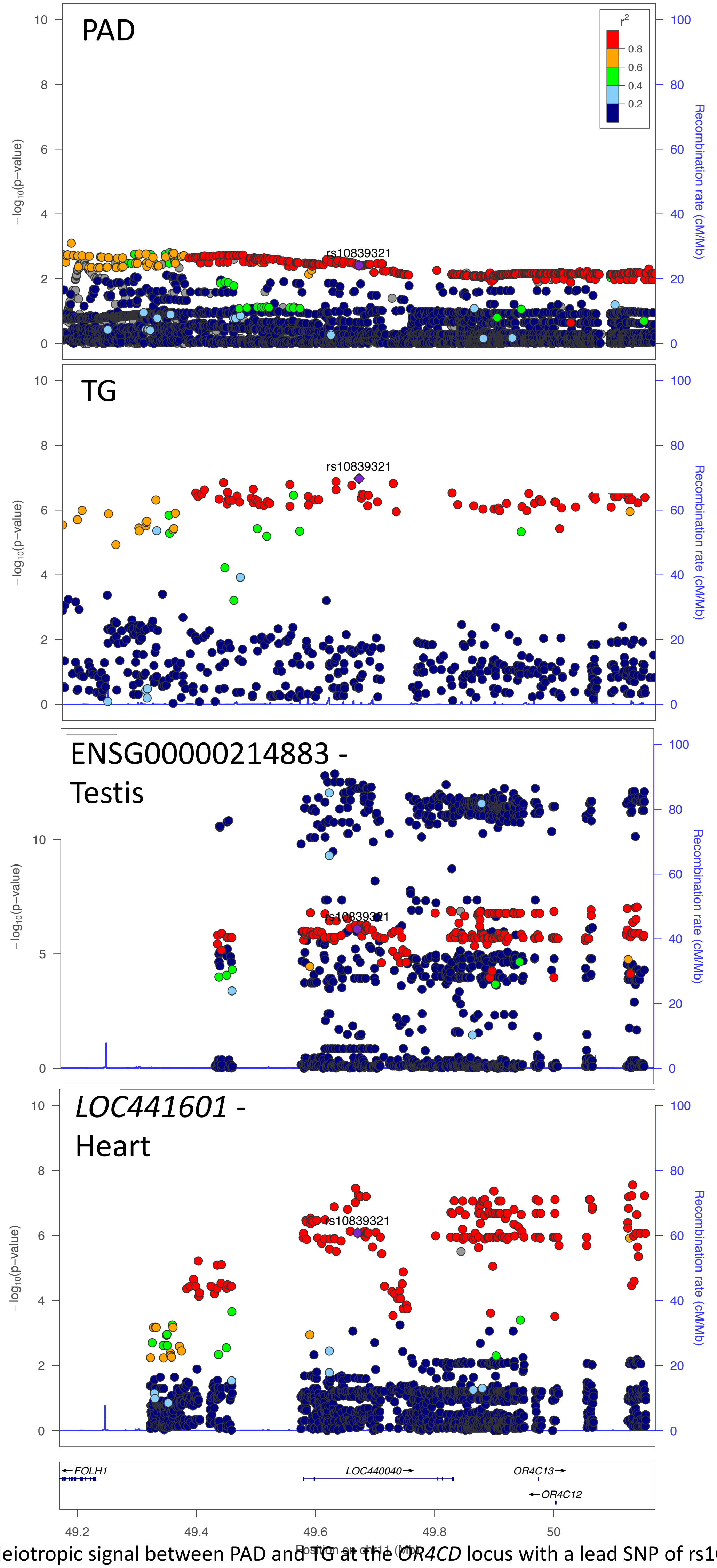

Figure S31. *OR4CD* locus. Pleiotropic signal between PAD and TG at the *OR4CD* locus with a lead SNP of rs10839321. The bottom 2 panels show the association peak for each eQTL detected from GTEx v8: ENSG00000214883 in testis tissue and *LOC441601* in heart left ventricle tissue.

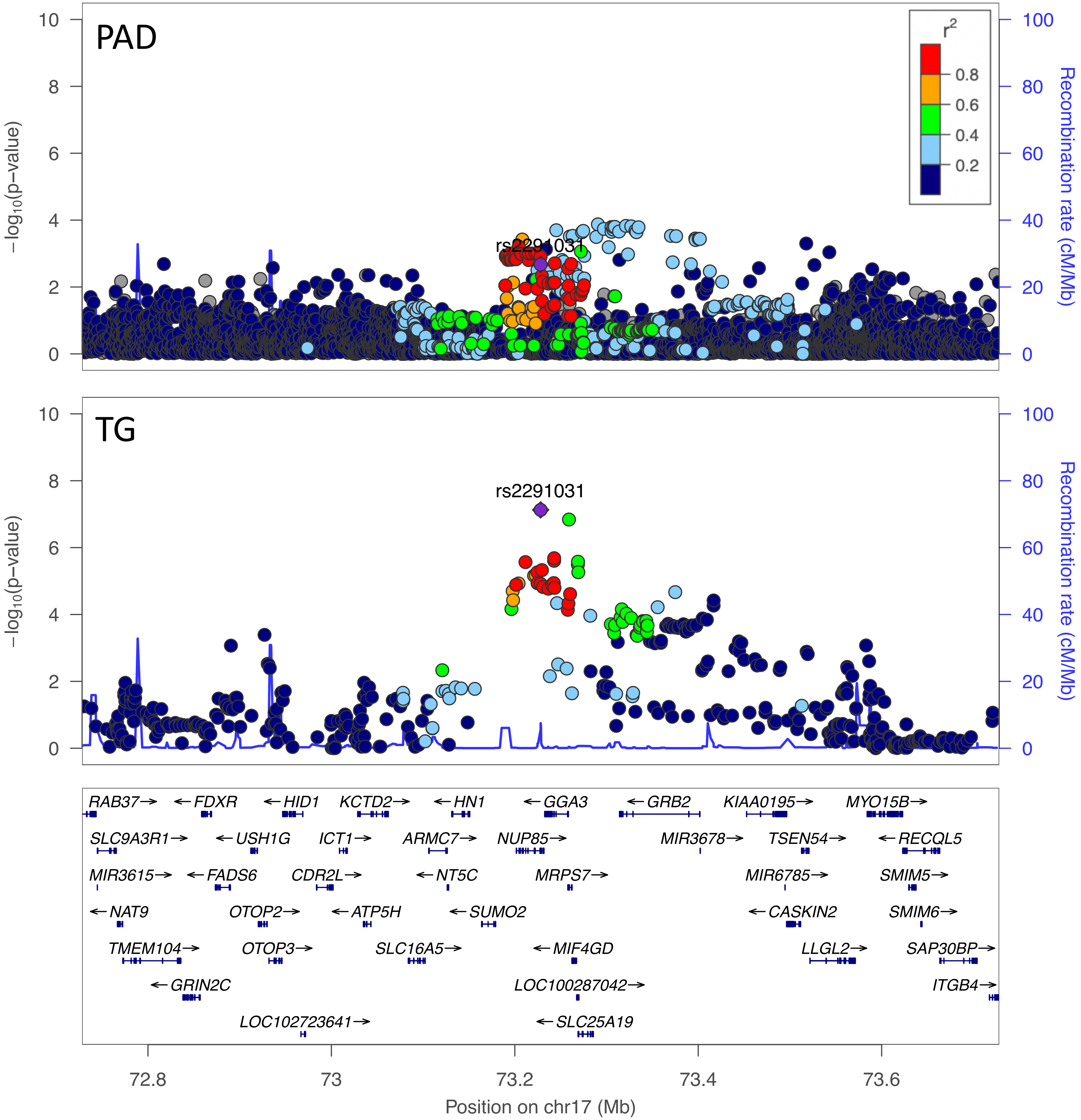

Figure S32. *NUP85* locus. Pleiotropic signal between PAD and TG at the *NUP85* locus with a lead SNP of rs2291031. Both PAD and TG data were conditioned on the SNPs rs141786503 and rs113613492 to achieve a conditional posterior probability of colocalization >0.8.

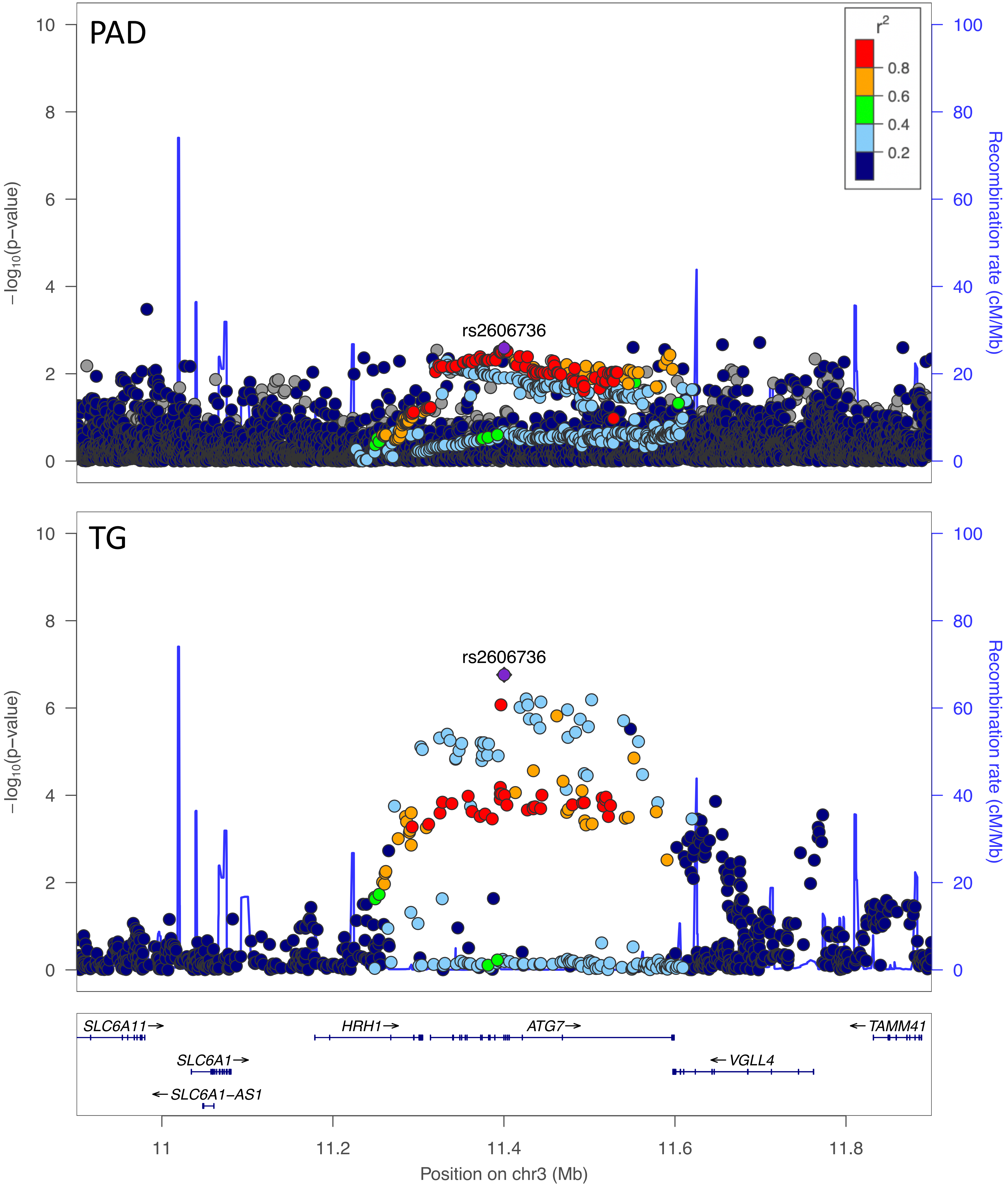

Figure S33. *ATG7* locus. Pleiotropic signal between PAD and TG at the *ATG7* locus with a lead SNP of rs2606736.

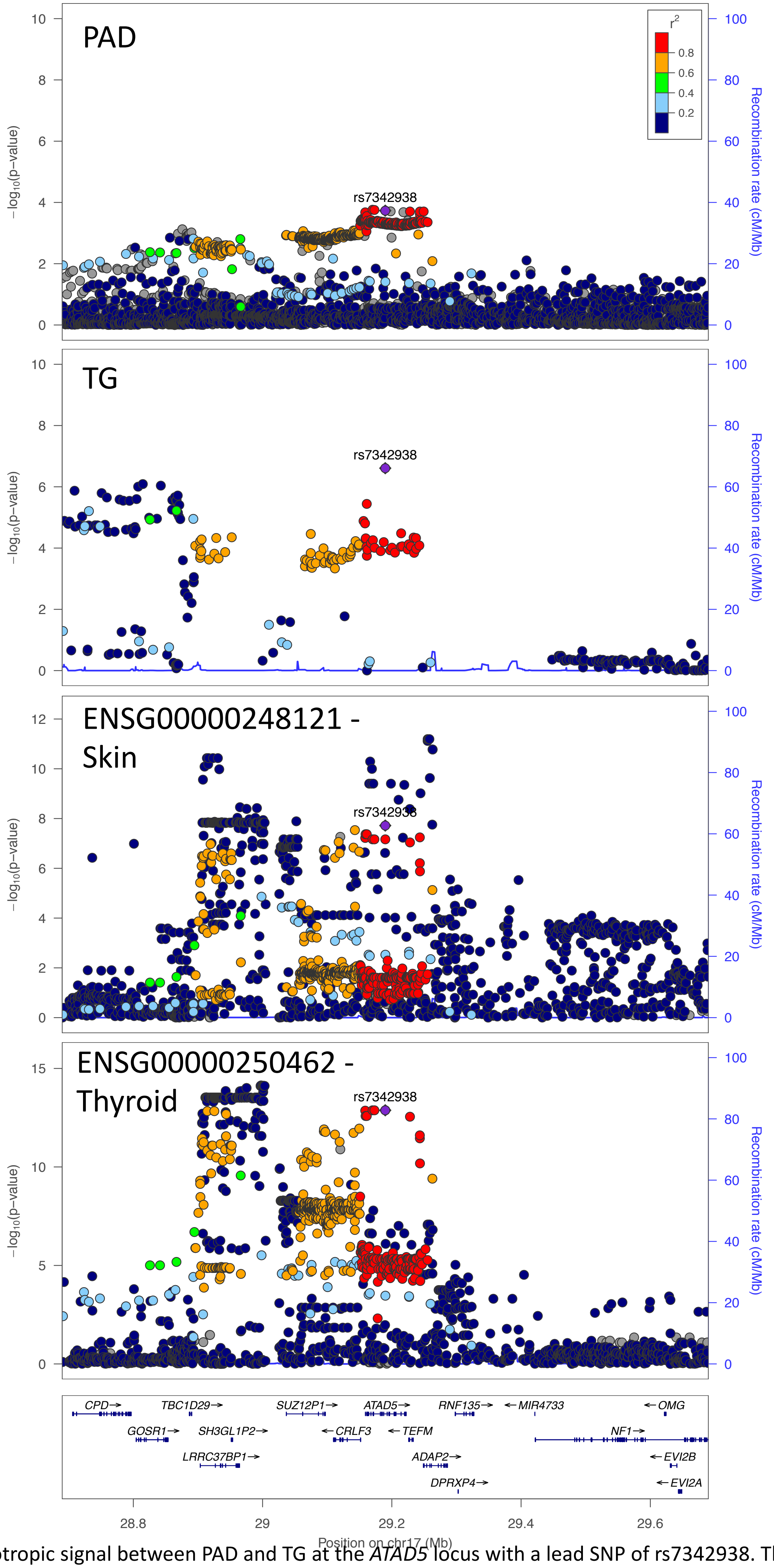

Figure S34. *ATAD5* locus. Pleiotropic signal between PAD and TG at the *ATAD5* locus with a lead SNP of rs7342938. The bottom 2 panels show the association peak for each gene eQTL detected from GTEx v8: ENSG00000248121 in suprapubic non-sun exposed skin and ENSG00000250462

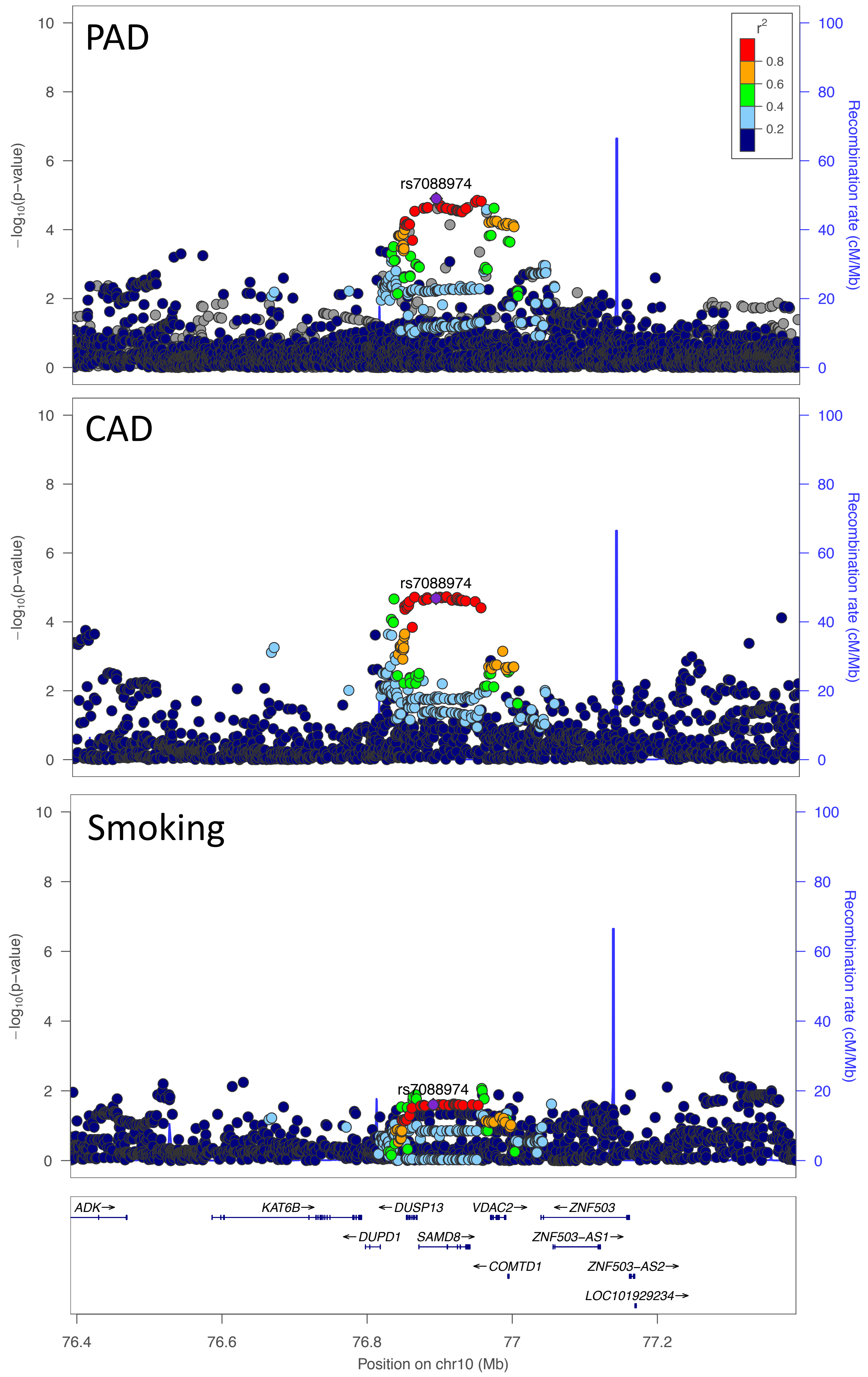

Figure S35. *VDAC2* locus pleiotropic signal between PAD and CAD has no evidence of smoking association.

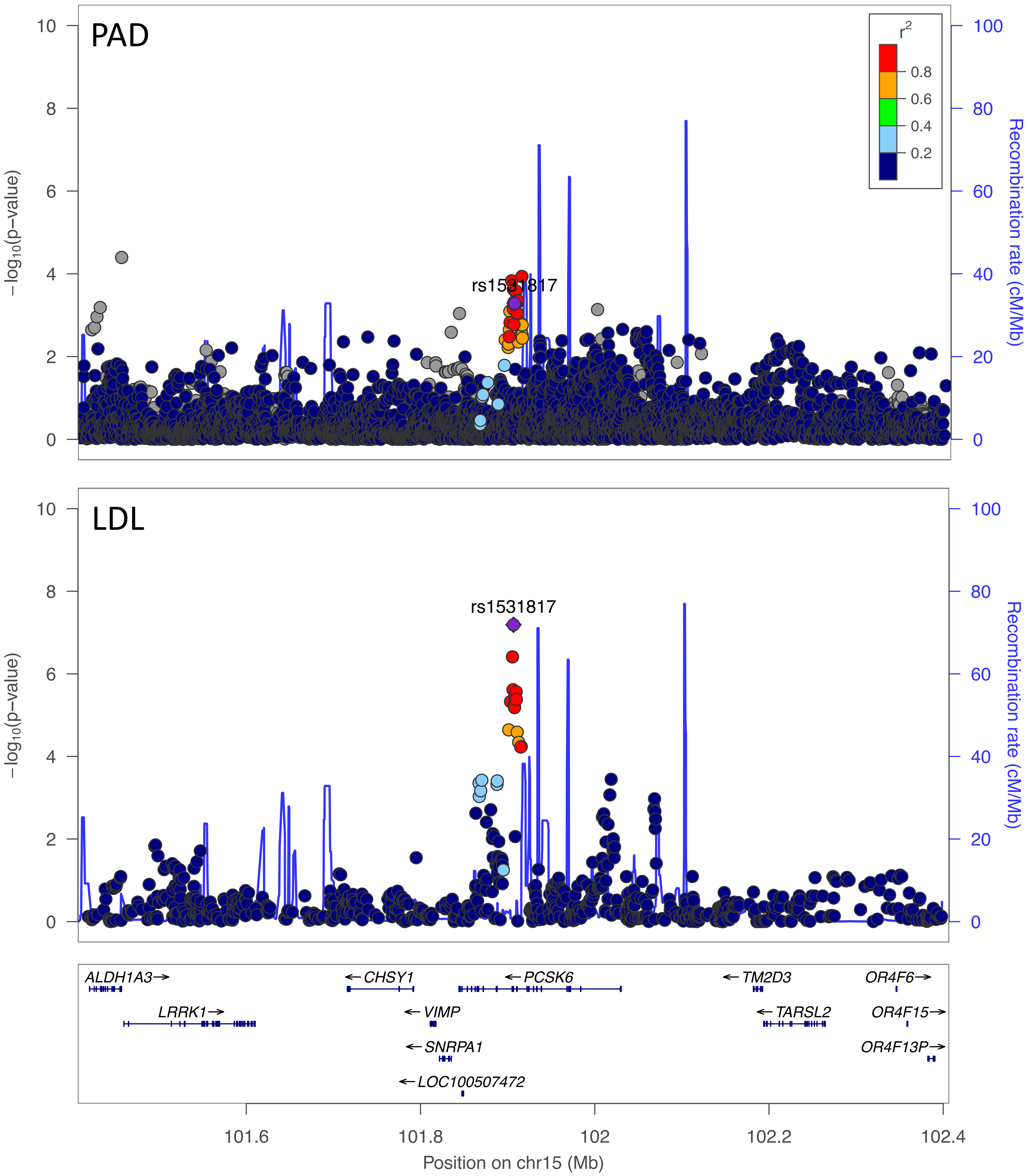

Figure S36. *PCSK6* locus pleiotropic signal between PAD and LDL conditioned on the recently identified coding variant rs34631529 in an LDL GWAS. The PAD signal was largely unchanged. This SNP was not present in LDL data.
